# Polymorphism of VDR Gene and the Sensitivity of Human Leukemia and Lymphoma Cells to Active Forms of Vitamin D

**DOI:** 10.3390/cancers14020387

**Published:** 2022-01-13

**Authors:** Justyna Joanna Gleba, Dagmara Kłopotowska, Joanna Banach, Eliza Turlej, Karolina Anna Mielko, Katarzyna Gębura, Katarzyna Bogunia-Kubik, Andrzej Kutner, Joanna Wietrzyk

**Affiliations:** 1 Department of Experimental Oncology, Hirszfeld Institute of Immunology and Experimental Therapy, Polish Academy of Sciences, Weigla 12, 53–114 Wroclaw, Poland; dagmara.klopotowska@hirszfeld.pl (D.K.); joanna.banach@hirszfeld.pl (J.B.); joanna.wietrzyk@hirszfeld.pl (J.W.); 2 Department of Experimental Biology, Wroclaw University of Environmental and Life Sciences, Norwida 27 B, 50-375 Wroclaw, Poland; eliza.turlej@upwr.edu.pl; 3 Department of Biochemistry, Molecular Biology and Biotechnology, Faculty of Chemistry, Wroclaw University of Science and Technology, Norwida 4/6, 50-373 Wroclaw, Poland; karolina.mielko@pwr.edu.pl; 4 Laboratory of Clinical Immunogenetics and Pharmacogenetics, Hirszfeld Institute of Immunology and Experimental Therapy, Polish Academy of Sciences, Weigla 12, 53–114 Wroclaw, Poland; katarzyna.gebura@hirszfeld.pl (K.G.); katarzyna.bogunia-kubik@hirszfeld.pl (K.B.-K.); 5 Department of Bioanalysis and Drug Analysis, Faculty of Pharmacy, Medical University of Warsaw, 1 Banacha, 02-097 Warsaw, Poland; andrzej.kutner@wum.edu.pl

**Keywords:** calcitriol, tacalcitol, leukemia, lymphoma, vitamin D receptor (VDR), 1,25D_3_–membrane-associated rapid response to steroids (1,25D_3_-MARRS), VDR gene polymorphism

## Abstract

**Simple Summary:**

Developing new therapeutic strategies is necessary for leukemias and lymphomas treatment. Therefore, the study aimed to determine the use of an active form of vitamin D_3_ calcitriol and its analog tacalcitol as an anticancer drug. Most of all, selecting the molecular factor responsible for the cell’s sensitivity to the tested agents. A biomarker that could be use in the future to select patients for therapy and improve survival outcomes. We examined nine cell lines and evaluated the proteins involved in the biological activity of calcitriol and tacalcitol: the classical vitamin D receptor and 1,25D_3_-MARRS, as well as polymorphism in the VDR gene receptor. Results showed that VDR polymorphism may predispose to response to calcitriol and tacalcitol anticancer therapy.

**Abstract:**

The active forms of vitamin D_3_ (calcitriol and tacalcitol) coupled to the vitamin D receptor (VDR) are known to exhibit anti-cancer properties. However, not all cancer cells are sensitive to the active forms of vitamin D_3_ and its analogs. The study aimed to determine whether polymorphism of VDR is responsible for the sensitivity of human leukemia and lymphoma cells to calcitriol and tacalcitol. The impact of calcitriol and tacalcitol on the proliferation and morphology of nine different leukemia and lymphoma cell lines was determined. Only MV-4-11, Thp-1, and HL-60 cell lines sensitive to proliferation inhibition by calcitriol and tacalcitol showed morphology changes. Subsequently, the levels of the VDR and 1,25D_3_-MARRS proteins of calcitriol and tacalcitol binding receptors and the VDR receptor polymorphism in human leukemia and lymphoma cells were ascertained. Contrary to the current understanding, higher levels of VDR are not responsible for the greater sensitivity of cells to calcitriol and tacalcitol. Importantly, we first showed that sensitivity to calcitriol and tacalcitol in leukemias and lymphomas could be determined by the VDR polymorphism. The *Fok*I polymorphism and the presence of the “bat” haplotype were observed only in the sensitive cells.

## 1. Introduction

Leukemia and lymphoma rank among the top 10 most common cancers with high mortality [1]. At present, myeloid and lymphoid cancer treatments are not very effective. Therefore, new therapies are needed to increase efficiency and tolerance compared to standard treatments [2,3]. Currently, attempts are being made to use the active form of vitamin D_3,_ calcitriol, and its derivatives (VDAs, vitamin D analogs) in patients with myeloid tumors. One of the very active and less calcaemic analogs of calcitriol (tacalcitol, PRI-2191) is used to treat hyperproliferative diseases such as psoriasis and vitiligo [4,5]. We have shown early in our studies [6], that the oral administration of the active forms of vitamin D to mice increased the calcium level by 78% for calcitriol and only to 47% over the control for tacalcitol. Numerous clinical trials with calcitriol have shown promising results in the treatment of myelodysplastic syndrome (MDS) and acute myeloid leukemia (AML) [7,8]. The appropriate cytogenetic and molecular classification was reported as a useful tool in selecting patients for differential therapies [9,10]. Therefore, it is necessary to identify biomarkers that would enable the selection of patients for cell differentiation therapy with vitamin D compounds. It is well documented that the VDR is responsible for the biological activity of vitamin D [11]. However, it turns out that calcitriol shows activity unrelated to VDR. Calcitriol has also a high affinity for the 1,25D_3_-MARRS (1,25-dihydroxyvitamin D_3_-membrane-associated rapid response to steroids) receptor. This protein is located mainly in the endoplasmic reticulum, cell membrane, cytoplasm, and the internal nuclear matrix [12,13,14]. Its presence and strong binding of calcitriol may affect the sensitivity of leukemia and lymphoma cells. Moreover, gene polymorphism of VDR may play an essential role in response to calcitriol. There are four well-described polymorphisms in the vitamin D receptor gene: *Fok*I (rs2228570) located in the second exon and related to the translation initiation site. The change of nucleotide from T (ATG) to C (ACG) causes a shift of the reading frame [15]. Three other polymorphisms, *BsmI* (rs1544410), *ApaI* (rs7975232), and *TaqI* (rs731236), in the 3′UTR region, have the most remarkable association. The 3′UTR region is responsible for the regulation of gene expression and especially for the stability of mRNA. The polymorphism within these regions leads to the formation of haplotypes responsible for activity, stability, and level of the native VDR [16]. Numerous studies indicate that there is a relationship between VDR polymorphism and the development of cancer [17]. However, there is no evidence of VDR polymorphism and the response to calcitriol and its analogs in leukemia and lymphoma cells. We first showed that it is neither the level VDR nor 1,25D_3_-MARRS, but the polymorphism of VDR that is crucial for calcitriol and tacalcitol biological activity. To fully elucidate the role of this polymorphism, it is essential to conduct additional in vivo studies and tests on patient-derived cells and xenografts.

## 2. Materials and Methods

### 2.1. Human Cell Lines

Human leukemia (KG-1, K562, HL-60, MV-4-11, Thp-1), and lymphoma (Jurkat, Daudi, Raji) cells were obtained from the American Type Culture Collection (ATCC, Rockville, MD, USA). U2932 was purchased from the Leibniz Institute DSMZ-German Collection of Microorganisms and Cell Cultures (Braunschweig, Germany). Cells were cultured in RPMI 1640 medium (Gibco, Scotland, UK) with 10% fetal bovine serum, 2 mM L-glutamine, 1.0 mM sodium pyruvate, 1.5 g/L sodium bicarbonate, and 4.5 g/L glucose (all from Sigma–Aldrich Chemie GmbH, Steinheim, Germany). K562 and Daudi culture medium was buffered with 20 mM HEPES (Sigma-Aldrich Chemie GmbH, Steinheim, Germany). All culture media were supplemented with 100 units/mL penicillin and 100 μg/mL streptomycin (both from Polfa Tarchomin S.A., Warsaw, Poland). Leukemia and lymphoma cells are maintained at the Hirszfeld Institute of Immunology and Experimental Therapy, Wroclaw, Poland. Cells were cultured in a humidified atmosphere with 5% CO_2_ at 37 °C.

### 2.2. Normal Human Blood

Peripheral blood samples (10 mL each) from 10 healthy people who consciously and voluntarily submitted them for testing and signed individual statements were collected after obtaining the consent of the Bioethics Committee at the Medical University of Wroclaw, No. 71/2017. Whole blood was collected in test tubes with an EDTA anticoagulant by specialized personnel of the diagnostics laboratory at the Institute of Immunology and Experimental Therapy. Blood mononuclear cells were then isolated using the Ficoll-Paque PLUS kit (GE Healthcare, Amersham, UK) according to the manufacturer’s instructions.

### 2.3. Compounds

Two vitamin D compounds were tested: calcitriol and tacalcitol (PRI-2191). Compounds were synthesized at the Pharmaceutical Research Institute (PRI, Warsaw, Poland). Solutions of all compounds were prepared in absolute ethanol to the final concentration of 10^−4^ M and stored at −20 °C. For in vitro examination, analogs were suspended in culture medium to level from 1 to 1000 nM. Since the vitamin D compounds are light-sensitive, their exposure to light was limited during all procedures. Ethanol (Avantor, Gliwice, Poland) was used as vehicle control and cisplatin (Teva Pharmaceuticals Poland, Warsaw, Poland) as a positive control. 

### 2.4. Anti-Proliferative Assay In Vitro

Cells (1 × 10^5^/mL) were suspended in a 10 mL culture medium and then seeded onto 96-well plates at 100 µL per well (Corning Incorporated, Corning, NY, USA). After 24 h, compounds were added in concentrations ranging from 1000 to 1 nM for 120 h. Next, 20 μL MTT (5 mg/mL) solution (Sigma–Aldrich, Steinheim, Germany) was added to each well and incubated for 4 h. After the incubation time, 80 μL of lysis buffer ((67.5 g sodium dodecyl sulfate SDS (Sigma–Aldrich, Steinheim, Germany), 225 mL N, N-Dimethylformamide (Avantor, Gliwice, Poland) and 275 mL MilliQ water IIET, Wroclaw, Poland)) were added to each well. After 24 h, a spectrophotometric measurement was carried out at 570 nm using a Synergy H4 Hybrid Multi-Mode Microplate Reader (BioTek Instruments, Inc., Winooski, VT, USA). Based on the absorbance value, the percentage of proliferation inhibition and the concentration of calcitriol and tacalcitol that causes 50% inhibition of cell proliferation-IC50 (IC50, inhibitory concentration 50) was determined using the Cheburator 0.4 Dmitry Nevozhay software. A minimum of 3 independent tests were performed.

### 2.5. Cell Morphology–Cytospin

Cells were collected from 10 cm dishes, washed with PBS (IIET PAS, Wrocław, Poland) with 2% FBS (Sigma–Aldrich, Steinheim, Germany). Centrifuged for 5 min, 400× *g* at 4 °C then the supernatant was removed. The cell pellet was suspended in 2 mL PBS and centrifuged again. Then 150 μL PBS was added, and the cells were placed on slides using a cytospin centrifuge (Shandon Cytospin, Thermo Scientific, Waltham, MA, USA) for 7 min at 700× *g* at room temperature. After centrifugation, the slides were allowed to dry at room temperature for 24 h. Next, cells were fixed for 20 min at room temperature with 100% ice-cold methanol (Avantor, Gliwice, Poland). After methanol evaporation, they were stained with May–Grunwald dye (Merck-Millipore, Darmstadt, Germany), diluted 1:1 in solution TRIS-HCl at pH 7.6 (IIET PAS, Wroclaw, Poland). Slides were rinsed in running water and placed for 15 min in a Hellendahl chamber filled with Giemsa’s reagent (Merck-Millipore, Darmstadt, Germany) diluted 1:9 in a TRIS-HCl solution pH 7.6. Samples were washed and allowed to dry at room temperature and viewed under a light microscope at 1000× magnification. Morphological analysis of the cells was performed using an Olympus CX41 light microscope (Olympus Europa Holding GMBH, Hamburg, Germany).

### 2.6. Cell Surface Markers

Surface antigen expression was carried out using the flow cytometry method. 2 × 10^5^ cells were suspended in a 100 μL PBS solution with 2% FBS, then incubated for 30 min with an antibody cocktail (Becton Dickinson, San Jose, CA, USA): against the following antigens (fluorescent dyes in brackets): MV-4-11, Thp-1, HL-60—CD11 (PE), CD14 (PerCP-Cy5.5) K562 and KG-1—CD34 (APC), CD38 (FITC), Jurkat—CD4 (BV450), CD8 (PE-Cy7), Raji Daudi, U2932—CD19 (BV421), and CD20 (Alexa 700). The isotype controls were used from the same vendor and at the same final concentrations for all tested antibodies. Cells were incubated for 30 min at room temperature in the dark and washed two times with PBS with 2% FBS. Fluorochrome conjugated with IgG1 antibody was used as a negative control. Analysis of surface antigens expression was performed using a BD LSR Fortessa II cytometer (Becton Dickinson, San Jose, CA, USA). The results were analyzed using Flowing Software version 2.5.1 (Cell Imaging Core, Turku Center for Biotechnology, Perttu Terho, Turkey).

### 2.7. Vitamin D Receptor and 1,25-D_3_-MARRS Protein Level Analysis Using Western Blot Method

Cells were washed in a PBS solution (IIET PAS, Wrocław, Poland) then 80 μL of a RIPA buffer (Sigma–Aldrich, Steinheim, Germany) containing inhibitors of proteases (Protease Inhibitor Cocktail, Sigma–Aldrich, Steinheim, Germany) and phosphatases (Phosphatase Inhibitor Cocktail 2 and Phosphatase Inhibitor Cocktail 3, Sigma–Aldrich, Steinheim, Germany) were added. Cells were incubated on ice for 15 min and then stored at −80 °C. Before measuring the protein concentration, the samples were centrifuged at 10,000× *g* for 10 min at 4 °C. The supernatant was transferred into new tubes. Determination of protein concentration was performed using the DC Protein Assay kit (Bio-Rad Laboratories, Hercules, CA, USA). Samples were diluted 30-fold in RIPA buffer. The absorbance was measured at 650 nm using a Synergy H4 Hybrid Multi-Mode Microplate Reader (BioTek Instruments, Inc., Winooski VT, USA). 4× Laemmli Sample Buffer (Bio-Rad Laboratories, Hercules, CA, USA) with 2-beta-mercaptoethanol (Sigma–Aldrich, Steinheim, Germany) was added to the samples with 30 μg of protein and these samples were heated for 5 min at 95 °C. The separated samples were then transferred to a 0.45 μm PVDF membrane (GE Healthcare, Amersham, Little Chalfont, UK). Protein transfer was carried out for 1 h in a semi-Dry Trans-Blot SD Semi-Dry Transfer Cells apparatus (Bio-Rad Laboratories, Hercules, CA, USA). The membrane was then blocked using a 5% blocking solution (Membrane Blocking Agent; GE Healthcare, Amersham) at 4 °C overnight. Membranes were incubated at room temperature with primary rabbit anti-VDR and anti-ERp57 (Santa Cruz Biotechnology Inc., Santa Cruz, CA, USA) diluted 1:500 and anti-β-actin (Sigma-Aldrich, Steinheim, Germany) diluted at 1:1000. Then membranes were incubated for 1 h with horseradish peroxidase-conjugated anti-rabbit antibodies (GE Healthcare, Amersham, UK) diluted at 1:10,000. The next step was incubation in the dark for 30 min with ECF substrate (GE Healthcare, Amersham, UK). Fluorescence intensity was measured using a Carestream Image Station 4000 MM PRO camera (Carestream Health, Woodbridge, CT, USA). Densitometric analysis was performed using ImageJ 1.46r software (National Institutes of Health, Bethesda, MA, USA). 

### 2.8. Detection of Vitamin D Receptor (VDR) Polymorphisms

Genomic DNA was extracted from human leukemia and lymphoma cell lines (3 × 10^6^ cells) using the NucleoSpin^®^ Blood Kit (Macherey-Nagel INC, Bethlehem, PA, USA). The amount and quality of DNA were estimated with the use of the NanoDrop 2000 spectrometer (ThermoFisher Scientific, Waltham, MA, USA). Vitamin D receptor gene polymorphisms were analyzed employing polymerase chain reaction (PCR) amplifications, followed by restriction fragment polymorphism length analyses (RFLP) with *Fok*I, *Bsm*I, *Apa*I, and *Taq*I restriction enzymes. The primers used for the PCR-RFLP assays were synthesized by the DNA Sequencing and Synthesis Laboratory (Institute of Biochemistry and Biophysics of the Polish Academy of Sciences, Warsaw, Poland). A minimum of 3 independent tests were performed. The primer sequences were as follows:

*Fok*I (rs2228570)
5′ AGCTGGCCCTGGCACTGACTCTGCTCT 3′
5′ ATGGAAACACCTTGCTTCTTCTCCCTC 3′
*Bsm*I (rs1544410)
5′CAACCAAGACTACAAGTACCGCGTCAGTGA′
5′ AACCAGCGGGAAGAGGTCAAGGG 3′
*Apa*I (rs7975232) and *Taq*I (rs731236)
5′-CAG AGC ATG GAC AGG GAG CAA G-3′
5′-GCA ACT CCT CAT GGC TGA GGT CTC A-3′

To determine VDR polymorphism, 50 ng of genomic DNA were used as a template for PCR amplification performed in a 20 μL reaction mixture containing, 1× concentrated polymerase buffer, 3 mM MgCl_2_, 0.2 mM dNTP, and 10 mM of each primer and Taq polymerase. PCR conditions were as follows: 94 °C for 5 min as an initial denaturation step, followed by 30 cycles at 94 °C for 30 s, at 64 °C for 30 s, at 72 °C for 45 s. Obtained PCR products were digested using fast *Fok*I, *Bsm*I, *Apa*I (37 °C for 1 h) and *Taq*I (65 °C for 30 min) enzymes. Restriction enzymes and buffers were purchased from Thermo Fisher Scientific, Waltham, USA. Digested fragments were separated on a 2% agarose gel (EURx, Gdańsk, Poland) with the addition of SimplySafe dye (EURx, Gdańsk, Poland). The following electrophoretical patterns characterized detected genotypes: *Fok*I FF (250 bp), Ff (250, 189, 61 bp), ff (189, 61 bp), *Bsm*I BB (815 bp), Bb (815, 586, 230 bp), bb (586, 230 bp), *Apa*I AA (744 bp), Aa (744, 530, 217 bp), aa (530, 217 bp), *Taq*I TT (744, 530 bp), Tt (744, 530, 217 bp), and tt (530, 217 bp). 

### 2.9. Statistical Analysis

Statistical analysis was performed using the Statistica 7.1 software (StatSoft Inc., Tulsa, OK, USA). Before choosing the test, the normality of the distribution was checked by Levene’s test. Data showing normal distribution were analyzed using the one-way ANOVA test. Non-parametric data were analyzed using a Kruskal-Wallis test. Results were of statistical significance where *p* < 0.05. The graphs were prepared using GraphPad Prism version 6.04 for Windows (GraphPad Software, La Jolla, CA, USA).

## 3. Results

### 3.1. Leukemia and Lymphoma Cells Respond to Calcitriol and Tacalcitol

**Figure 1:**
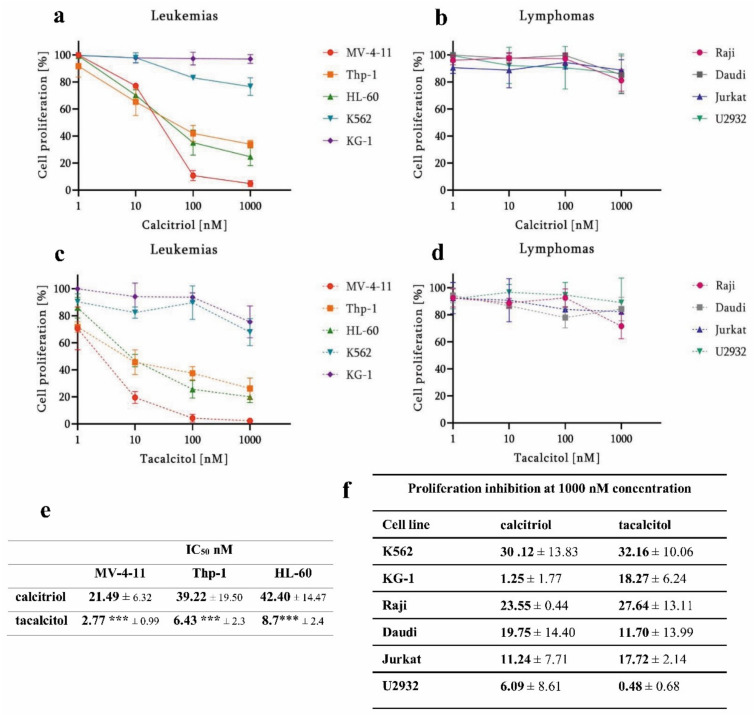
In vitro antiproliferative activity of calcitriol and tacalcitol against myeloid cancer cells. 1–1000 nM calcitriol (**a**,**b**) and tacalcitol (**c**,**d**) dose-out after 120 h exposure. Three cell lines: MV-4-11 acute myelomonocytic leukemia, Thp-1 acute monocytic leukemia and HL-60 acute myelocytic leukemia, showed high sensitivity to calcitriol and tacalcitol. The IC_50_ value of tested compounds in nM concentration (mean ± standard deviation). *** *p* < 0.05 as compared to calcitriol; Statistical analysis: Kruskal–Wallis multiple comparison test (**e**). The percent inhibition of proliferation after 1000 nM treatment is presented (mean ± standard deviation) (**f**).

The MTT proliferation assay was used to determine leukemia and lymphoma cells’ response to calcitriol and tacalcitol. The results showed that myeloid cancer cells were significantly more sensitive to calcitriol and tacalcitol compared to lymphoid cancer cells (Figure 1a–d).

In this study, nine leukemia and lymphoma cell lines were used. Only for three was it possible to determine the concentration that inhibits proliferation of 50% cells (IC_50_), these cells were classified as sensitive to calcitriol and tacalcitol. The most sensitive to the tested compounds were MV-4-11, Thp-1, and HL-60 leukemia cells. The highest anti-proliferative activity was observed in myelomonocytic biphenotypic leukemia cells MV-4-11 (AML-M5). The highest anti-proliferative activity was observed in myelomonocytic biphenotypic leukemia cells MV-4-11 (AML-M5). The IC_50_ value was as low as 3 nM for tacalcitol and 21 nM and calcitriol. Slightly lower activity was observed in acute monocytic leukemia cells Thp-1 (AML-M5): 6 nM tacalcitol, 40 nM calcitriol. In the case of acute promyelocytic leukemia cells HL-60 (AML-M2), the IC_50_ value for tacalcitol was 8 nM and calcitriol 42 nM (Figure 1e).

It was impossible to determine the IC_50_ value for the other two cancer cell lines of myeloid origin: acute myeloid leukemia KG-1 and chronic myeloid leukemia K562, as well as for all lymphoid cancer cells. For the remaining cell lines, the inhibition of proliferation at the highest used concentration of calcitriol and tacalcitol (1000 nM) was calculated. Among the two myeloid cancer cell lines K562 and KG-1, the highest proliferation inhibition was observed in chronic myeloid leukemia cells K562 (AML-M0). Both calcitriol and tacalcitol caused about 30% inhibition of cell proliferation. In acute myeloid leukemia KG-1 (AML-M1) cells, tacalcitol showed higher anti-proliferative activity (approx. 18%) compared to calcitriol (approx. 1%). 

In lymphoid cancer cells, the highest cell proliferation inhibition was observed against Burkitt’s lymphoma cells, Raji. Tacalcitol showed slightly higher inhibition activity (approximately 27%) than calcitriol (approximately 23%). The lower activity of used compounds was observed in lymphoma Daudi cells. The use of 1000 nM calcitriol inhibited about 20% of cells, while the use of tacalcitol inhibited about 12%. In T lymphocyte acute leukemia Jurkat cells, proliferation was inhibited by approximately 17% (calcitriol) and 11% (tacalcitol). The most insensitive cells were diffuse large B-cell lymphoma U2932 (Figure 1f). The solvent for the test substances was 99.8% ethyl alcohol, while the positive control was the standard of care drug cisplatin (Tables S1 and S2).

Tested cell lines were characterized in Table 1. Cell lines were listed according to the degree of differentiation based on the FAB (French–American–British) classification. In addition, the type of disease, sex, and age of the patient, doubling time, known gene fusions, and gene mutations have been specified. Data were collected from the Cellosaurus database.

### 3.2. Cell Morphology

**Figure 2:**
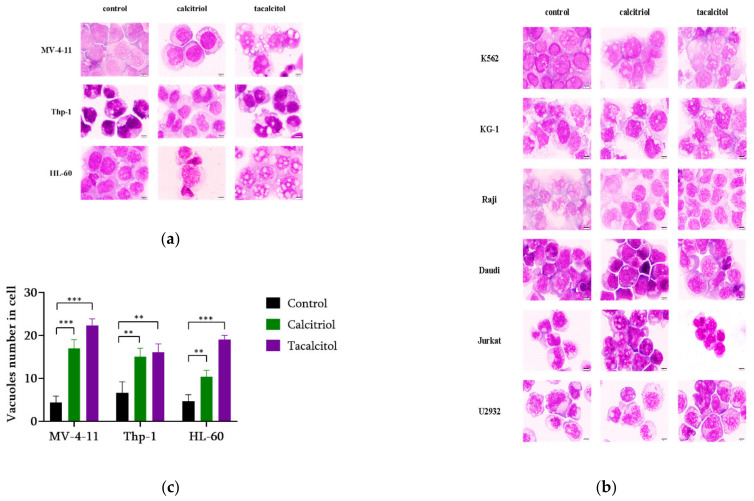
Human leukemia cells. Sensitive to calcitriol and tacalcitol MV-4-11, Thp-1, and HL-60 cells showed changes in cell morphology and numbers of vacuoles (**a**,**b**). Other lymphoma and leukemia cells were insensitive to calcitriol and tacalcitol. There were no changes in the morphology of these cells (**c**). May–Grunwald, Giemsa staining (the scale corresponds to 5 μm). Vacuole numbers (mean ± standard deviation). ** *p* <0.05 *** *p* <0.01 statistical significance to the control.

The response to calcitriol and tacalcitol prompted an effort to determine whether these substances affect leukemia or lymphoma cell morphology. It turned out that only the MV-4-11, Thp-1, and HL-60 cell lines which were sensitive to calcitriol and tacalcitol showed morphology changes. Untreated MV-4-11 cells were characterized by a large nucleus occupying about 75% of the cell volume, without any signs of brightness. No visible granularity or the presence of vacuoles was observed in the light blue cytoplasm. Cells treated with calcitriol and tacalcitol increased the volume ratio of the cytoplasm to the nucleus. The clearing zone around the nucleus and intensive vacuolization in tacalcitol treated cells were observed. Thp-1 cells were often characterized by a different-shaped nucleus, often resembling a “kidney” or “horseshoe” with a delicate chromatin structure located in the center of the cell. Before applying the tested compounds to Thp-1 cells, the ratio of nucleus to cytoplasm volume was around 1:1. After 120 h of stimulation with calcitriol and tacalcitol, an increase in cytoplasm volume was observed. Additionally, the appearance of numerous vacuoles after treating cells with tacalcitol was noticed. As with MV-4-11 cells, numerous large vacuoles were observed and were much more abundant in the cells treated with tacalcitol than calcitriol. Similar changes in cell morphology were noticed in HL-60 cells. A larger nucleus characterized non-treated cells. After using calcitriol and tacalcitol, the color of the cytoplasm changed from light blue to pale blue, with a visible zone of brightness around the nucleus. The cytoplasm contained numerous vacuoles, which were a larger size than those found in the cells stimulated with tacalcitol (Figure 2a,b). An increased number of vacuoles has been observed in cells sensitive to calcitriol and tacalcitol. Changes were statistically significant compared to control cells (Figure 2c).

K562 and KG-1 cells showed no change in morphology after treatment with calcitriol and tacalcitol. The cell nucleus’ shape and location and the ratio of nucleus volume to the cytoplasm were similar. There was no increase in the degree of cytoplasmic vacuolization. Moreover, lymphoid tumor cells used in the research showed no change in morphology in the microscopic image after using calcitriol and its analog tacalcitol (Figure 2b).

### 3.3. Analysis of Surface Markers

**Figure 3:**
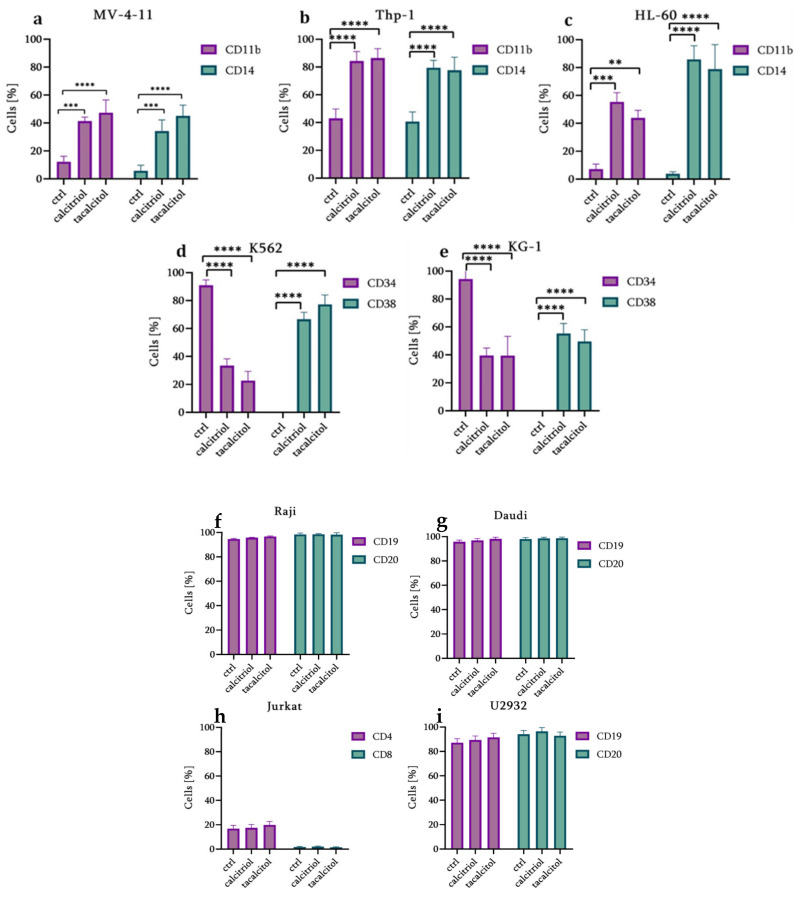
CD11b and CD14b antigens in MV-4-11 (**a**), Thp-1 (**b**), and HL-60 (**c**) cells sensitive to calcitriol and tacalcitol. CD34 and CD38 antigen levels in K562 (**d**) chronic myeloid leukemia and KG-1 (**e**) acute myeloid leukemia cells. CD19 and CD20 antigens in the cells of Burkitt Raji lymphoma (**f**), Burkitt Daudi lymphoma (**g**) and diffuse large B-cell lymphoma U2932 (**i**). CD4 and CD8 antigens levels in acute leukemia cells from Jurkat T cells (**h**). Analysis of surface antigens level after 120 h of incubation with 10 nM calcitriol and tacalcitol (mean ± standard deviation). ** *p* < 0.05, *** *p* < 0.01, **** *p* < 0.0001 statistical significance to the control.

Knowing that calcitriol and tacalcitol affect cells’ morphology, it was decided to assess the level of surface antigens characteristic for selected cell lines and verify whether calcitriol and its analog affect these proteins, and thus the process of cell differentiation. The level of the two surface markers characteristic of a selected cell was examined. The following surface antigens were selected for analysis: for myeloid tumors: MV-4-11, Thp-1, HL-60—CD11b, CD14, K562 and KG-1—CD34, and CD38; for lymphoid neoplasms: Raji, Daudi, U2932—CD19, CD20, Jurkat—CD4, CD8. The conducted analyses showed that 120 h incubation of myeloid tumors with calcitriol and tacalcitol causes significant changes in the tested antigens’ level. It was observed that in the MV-4-11, Thp-1, and HL-60 cells sensitive to calcitriol and its analog, CD11b and CD14 antigens increased (Figure 3a–c). Both calcitriol and tacalcitol increased the level of CD11b by about 30% in MV-4-11 cells. The level of CD14 also increased: tacalcitol increased the amount of this protein by 40%, and calcitriol by 30% compared to control cells (Figure 3a). Similar effects of calcitriol and tacalcitol were observed for cells of the second sensitive line—Thp-1. The tested substances increased CD11b and CD14 protein levels by about 40% compared to control cells. No differences in effectiveness were observed between the tested substances (Figure 3b). Treatment of HL-60 cells with calcitriol and tacalcitol also increased CD11b levels by approximately 40% compared to the control. However, significantly greater activity was noted for the CD14 protein, the level of which increased by about 80% compared to the control (Figure 3c). For chronic (K562) and acute (KG-1) myeloid leukemia cells, the CD34, and CD38 antigens were selected for analysis. It turns out that in both cell lines, over 90% of cells were characterized by the presence of the CD34 antigen, and no cells with the CD38 antigen had been observed. Interestingly, stimulation with calcitriol and tacalcitol resulted in a significant increase in CD38 levels and decreased CD34. Both calcitriol and tacalcitol increased the amount of the CD38 antigen by approximately 70% in K562 cells compared to the control (Figure 3d). A slightly lower effect of calcitriol and tacalcitol was observed in the case of KG-1 cells. The tested vitamin D derivatives increased the CD38 level by about 50% compared to control cells (Figure 3e). The isotype control antibodies for CD34 and CD38 markers were presented in Supplementary Materials Figure S3. The level of CD19 and CD20 antigens was determined in lymphoid neoplasms: Burkitt Raji, Daudi lymphomas, and diffuse large B lymphoma U2932. It turns out that in all cells derived from the B cell line, the level of CD19 and CD20 antigens was over 90%. Calcitriol and tacalcitol did not change CD19 and CD20 levels in these cells (Figure 3 f,g,i). CD4 and CD8 antigens were analyzed in a Jurkat T cell. Experiments showed about 16% of CD4 and 1% of CD8 antigens. Incubation with calcitriol and tacalcitol did not change the levels of these markers (Figure 3h).

### 3.4. Assessment of Calcitriol and Tacalcitol Binding Receptors in Human Leukemia and Lymphoma Cells

**Figure 4:**
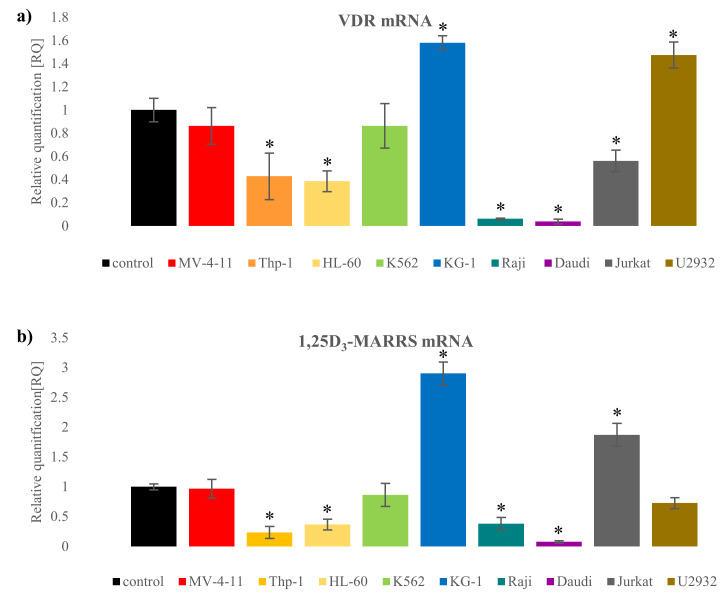
mRNA level of vitamin D binding receptors: classical vitamin D receptor (VDR) (**a**) and 1,25D_3_-MARRS (**b**) in human leukemia and lymphoma cells. The mRNA level was compared to the control level of normal blood cells. *—statistical significance (*p* < 0.05) in relation to the control.

Knowing that the vitamin D binding receptors: native VDR and 1,25D_3_-MARRS were responsible for the biological activity of calcitriol and tacalcitol, mRNA and protein levels were analyzed. The level was compared to the normal mononuclear blood cells. The most sensitive to vitamin D analogs MV-4-11 cells have similar mRNA levels of the classic vitamin D receptor and 1,25D_3_-MARRS compared to the level observed in normal blood mononuclear cells. In contrast, the other two sensitive cell lines, Thp-1 and HL-60, were characterized by a lower mRNA level of both receptors compared to the control cells. Among the other two myeloid cell lines, similar mRNA levels of both receptors in the K562 cells were observed to those in control cells. In contrast, KG-1 cells had a significantly higher mRNA level of both molecules tested than normal cells. The Raji and Daudi lymphoma cells had significantly lower mRNA levels of both receptors compared to normal blood cells. In turn, Jurkat cells had a lower level of mRNA for the VDR, but a higher level of 1,25D_3_-MARRS receptor mRNA compared to control cells. The U2932 cells were characterized by a higher level of VDR mRNA and a similar level of 1,25D_3_-MARRS mRNA to that observed in normal cells. Figure 4 mRNA level of VDR (a) and 1,25D_3_-MARRS (b).

**Figure 5:**
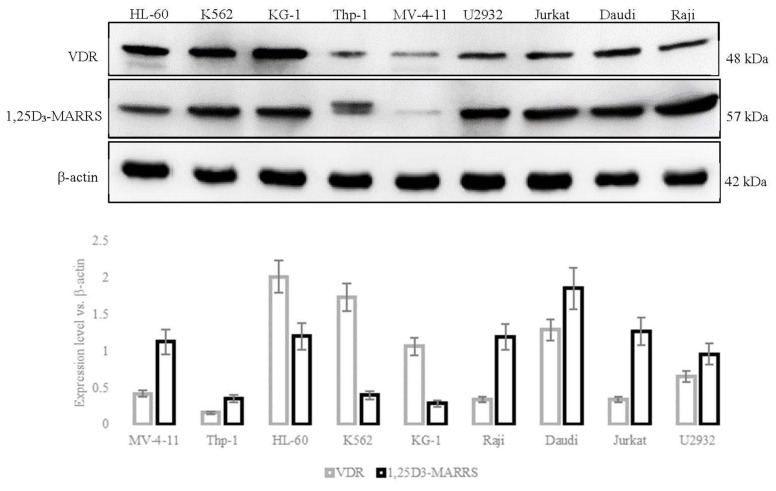
Protein levels of vitamin D-binding receptors: native VDR and 1,25D3-MARRS in leukemia and lymphoma cells. Relative protein levels were estimated by densitometric measurements of VDR and 1,25D_3_-MARRS signals obtained by Western blotting, were normalized to β-actin (representative of Western blotting results above the plot). Whole Western blots result presented in Figure S1a.

The evaluation of the level of protein of the examined receptors showed that the sensitive cell lines MV-4-11 and Thp-1 had a higher level of 1,25D_3_-MARRS protein compared to the VDR. In turn, the cells of the HL-60 line had a significantly higher level of VDR than 1,25D_3_-MARRS. In the other two K562 and KG-1 myeloid tumor cells the level of VDR was higher compared to 1,25D_3_-MARRS. Higher levels of 1,25D_3_-MARRS was observed in all lymphoid tumor cells insensitive to test substances compared to VDR (Figure 5).

**Figure 6:**
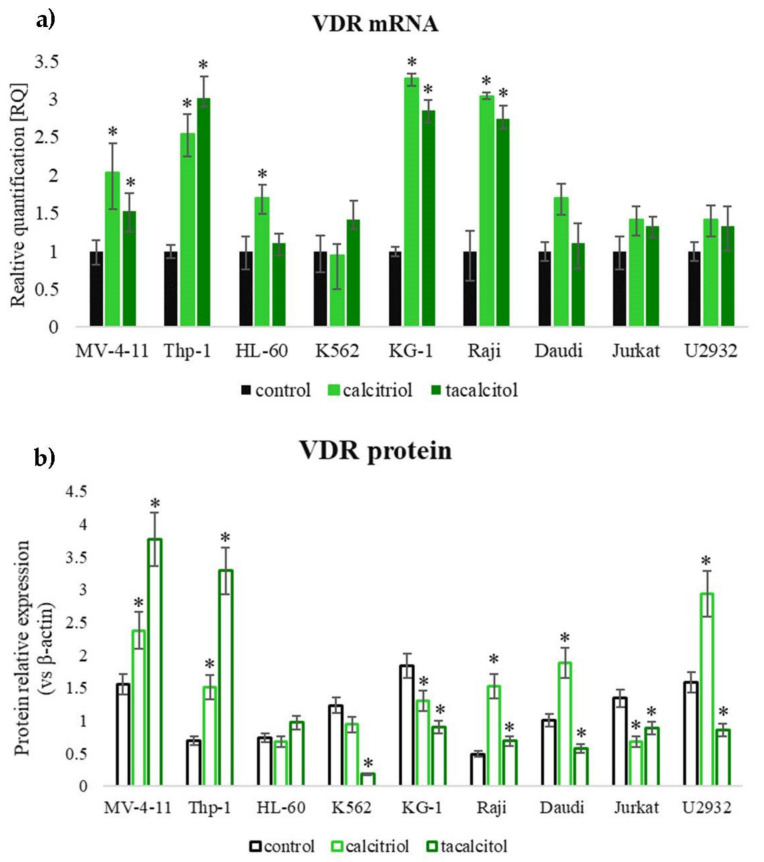
VDR mRNA (**a**) and protein (**b**) level after 120 h of treatment with calcitriol and tacalcitol. *—statistical significance (*p* < 0.05) compared to the control. Presented data from three independent experiments.

The level of mRNA and protein after incubation with calcitriol and tacalcitol was also examined. Cells were incubated for 120 h with vitamin D analogs. Interestingly, MV-4-11, Thp-1, and HL-60 VDR mRNA levels increased after treatment in all three sensitive cell lines. A similar effect was observed in two insensitive cell lines, KG-1 and Raji. Other tested cell lines did not show the changes in VDR mRNA level (Figure 6a). The VDR protein level analysis showed that calcitriol caused a significant increase in the level in both sensitive cells MV-4-11, Thp-1, and insensitive cells Raji, Daudi, and U2932. Interestingly, no changes in the level of VDR in sensitive HL-60 cells were observed after calcitriol and tacalcitol treatment (Figure 6b). We did not observe any correlation in the change in the level of VDR mRNA and protein compared to inhibition of proliferation (Western blots and densitometry analysis after 24, 48, 72, 96, and 120 h of treatment placed in Supplementary Materials (Figure S1b,c)).

### 3.5. Polymorphism of Vitamin D Receptor Gene in Human Leukemia and Lymphoma Cells

VDR polymorphism studies were performed to determine *Fok*I polymorphism, which can lead to the presence of three polymorphic variants: homozygotes (FF), one VDR protein variant 427 amino acids long, less transcriptional, and less responsive to calcitriol; heterozygotes (Ff)–two VDR protein variants, 427 amino acids long and 424 amino acids short, the combination of less and more transcriptional active forms of VDR, homozygotes (ff), only the shorter variant (424 aa) of the vitamin D receptor protein, that is more transcriptionally active. Addtionally, the *Bsm*I-*Apa*I-*Taq*I polymorphism was analyzed for the presence of “baT” haplotype, which is responsible for the high translational activity of VDR.

#### 3.5.1. *Fok*I Polymorphism

The *Fok*I polymorphism located in exon 2 of the VDR gene was examined to find out whether the f polymorphic variant, responsible for the formation of the shorter and more transcriptionally active form of the vitamin D receptor protein, is present in the myeloid and lymphoid cells. The analysis showed that cells sensitive to calcitriol and tacalcitol: MV-4-11, Thp-1, HL-60 cell lines, and insensitive Jurkat cell line were positive for the presence of the f allele at the translation initiation site of the VDR gene. These cells turned out to be heterozygous (Ff). For the two insensitive cell lines, K562 and KG-1, a cleavage site in the coding sequence for the vitamin D receptor gene by *Fok*I was also observed. These two cell lines were found to be recessive homozygotes (ff), producing only the shorter variant (424 aa) of the vitamin D receptor protein, that is more transcriptionally active. As for the *Fok*I polymorphism of the Raji, Daudi, and U2932 cells, they appeared to be dominant homozygotes (FF), which means that they produce only one protein variant 427 amino acids long, less transcriptional, and less responsive to calcitriol.

#### 3.5.2. *Bsm*I-*Apa*I-*Taq*I Polymorphism

**Figure 7:**
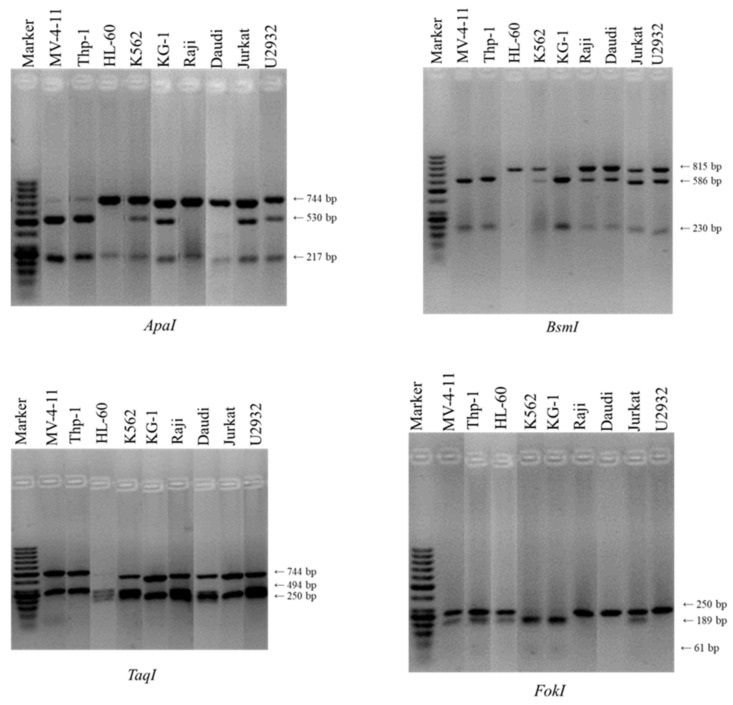
Polymorphism of vitamin D receptor. Alleles are shown of human leukemia and lymphoma cells in the gene coding for the vitamin D receptor. Above are examples of RLFP patterns (M-marker mass, numbers from 1–9 cell lines, names are given in the table). For easier tracking of the results, the gel photos were cut and arranged in order of lines responding and not responding to the use of calcitriol and tacalcitol. Gels before processing are presented in Figure S2.

In the next step, three VDR gene polymorphisms-*BsmI-ApaI-TaqI*-located within the 3′UTR region were analyzed to assess the presence of the “bat” haplotype, which determines the stability and translational activity of the vitamin D receptor mRNA. The study showed that two of the sensitive to the action of calcitriol and tacalcitol MV-4-11 and Thp-1 cell lines were characterized with the “baT” haplotype, which is associated with the higher translational activity of vitamin D receptor mRNA, resulting in the formation of more VDR protein in cells. At the same time, this haplotype is responsible for less transcript stability in the cell environment. In HL-60 cells the presence of the “BAt” haplotype was identified. It was the only cell line selected for research that had such a haplotype. Interestingly, all cell lines of Raji, Daudi, Jurkat, and U2932 lymphoid origin as well as K562 chronic myeloid leukemia cells were characterized by the presence of the “BAT” haplotype. In contrast, KG-1 cells had the “bAT” haplotype. The presence of these polymorphisms does not affect the stability and translational activity of vitamin D receptor mRNA. Determined polymorphism are shown in Figure 7, and haplotypes are summarized in Table 2. 

## 4. Discussion

An understanding of the molecular mechanisms of action of calcitriol and its analogs is crucial to developing new therapeutic strategies. Cancer cells respond to calcitriol and its analogues to a different extent. Indication of a biomarker that enables the appropriate selection of patients for treatment is also crucial in developing therapeutic strategies. Disorders of crucial stages of hematopoiesis are the leading causes of hematopoietic and lymphoid malignancies. Currently, these cancer types include over one hundred disease [18,19]. Among them, the expression of transcription factors is most commonly affected, as well as protein mutations responsible for epigenetic modifications. Additionally, genetic predisposition associated with the disorders of the mechanisms of DNA repair, cell cycle, and differentiation mechanisms underlie their pathogenesis. No less important are chromosomal translocations leading to the formation of fusion genes, as well as aneuploidy and unbalanced chromosomal aberrations [20,21]. The expectations associated with calcitriol’s ability to differentiate cells did not bring convincing results in early clinical trials [22]. It turned out that a therapeutic effect is associated with the use of hyper-physiological concentrations that lead to hypercalcemia, caused by the sudden release of calcium from the bone, which in turn, leads to osteomalacia and calcification of soft tissues [23]. Therefore, the synthesis of vitamin D analogs, provided compounds with similar or even higher anti-tumor activity, while low calcemic. In vitro studies using vitamin D analogs confirm their more significant anti-tumor activity and higher ability to induce cell differentiation in many types of cancer [24]. After discovering that vitamin D has anti-proliferative and pro-differentiation activities against cells expressing VDR, it was found that vitamin D can be used in therapy against other hematological malignancies, such as the lymphoid cancer types: Hodgkin’s lymphoma, non-Hodgkin’s lymphoma, and myeloma. However, the hypercalcemia that is often characteristic of Hodgkin’s and non-Hodgkin’s disease is also found to be mediated by calcitriol, leading to greater mortality and morbidity. Despite this paradox, vitamin D and its derivatives have become a subject of investigation in efforts to find suitable therapies against lymphoid malignancies. The mechanisms by which vitamin D could confer protection against Hodgkin’s and non-Hodgkin’s lymphoma were studied through the examination of VDR polymorphisms in human populations. Single nucleotide polymorphisms in *Fok*I, *Bsm*I, and *Taq*I restriction sites in the VDR gene were not found to have a clear relationship with risk for NHL and VDR polymorphism. However, the study did find certain *Bsm*l and *Taq*I alleles to be associated with increased risk for diffuse large B-cell lymphoma and a *Fok*I allele to be related to increased risk for T cell lymphoma, possibly due to decreased transactivation of VDR [25]. While the relationship between VDR polymorphism and lymphoid malignancies has not been firmly established, vitamin D has been found to exert anti-proliferative and differentiating effects on VDR-expressing lymphocytes and lymphocytic precursors. However, the effects of clinical trials that have been conducted so far (mainly patients with AML, but also with NHL) seem to depend on the individual characteristics of a particular patient, not only on the disease. Interesting ex vivo research was conducted by Baurska et al. [26] and Gocek et al. [9] using blasts from AML patients. It showed how differentiated the response to the action of calcitriol analogs is between patients with AML. Thus, based on studies conducted on a limited number of cell lines, it is not possible to draw unequivocal conclusions [27].

Reported studies have shown that tacalcitol caused more significant inhibition of proliferation compared to calcitriol, while being, beneficially, less calcaemic. The biological activity of the tested compounds was analyzed concerning the differentiation status according to the FAB classification. It was observed that only cells in the M2, M5 differentiation stage (the most differentiated cells) were sensitive to tested agents. The highest anti-proliferative activity of the active forms of vitamin D was observed in myelomonocytic biphenotypic leukemia cells MV-4-11 (AML-M5). As expected, the IC_50_ value for tacalcitol was much lower (3 nM) than that of calcitriol (21 nM), demonstrating that tacalcitol might be a safer potential anticancer therapeutic than calcitriol. Among myeloid neoplasms, the highest activity of the tested substances was observed against M5 type MV-4-11, Thp-1 cells. The exception was the HL-60 cells, which are classified according to the division of FAB into type M2, at the same time this type of acute leukemia is defined as a good prognosis. For the other two cell lines (K562 and KG-1) M1 and M0, such high activity of calcitriol and its analogue were not observed. Similarly, in the case of lymphoid-derived tumors, the higher inhibition of proliferation was observed in Burkitt’s lymphoma cells, which in the FAB classification belong to the L3 subtype [28]. Only sensitive cells showed changes in their morphology, leading to an increase in the degree of cytoplasmic vacuolation, which could be a hallmark of the autophagy process [29]. Initiating the autophagy process sensitizes breast cancer cells MCF-7 to ionizing radiation. Vitamin D liganded VDR, induces autophagy in mammary gland cancer cells, which correlates with increased survival in patients [30]. 

It turns out that the basal level of VDR and 1,25D_3_-MARRS mRNA and protein does not allow for the selection of cells sensitive to vitamin D analogs. Examination of the level of the two receptors: the native VDR and the 1,25D_3_-MARRS capable of binding calcitriol and tacalcitol showed that the best effect of the tested substances was observed when the level of both tested proteins increased under their influence Interestingly, cells insensitive to vitamin D analogs also showed elevated VDR levels, which did not cause a biological response. The research suggests that the type and biological activity of VDR determines the sensitivity to calcitriol and tacalcitol. Until recently, it was thought that only one vitamin D receptor-VDR was responsible for the biological activity of calcitriol and its analogs in cells [31]. This protein is a transcription factor that, after ligand activation, binds to the elements of vitamin D response (VDREs) in target gene promoters causing either their activation or blocking of gene expression [32]. It has been proven that the presence of the classic vitamin D receptor-VDR in keratinocytes is necessary for the proper growth and proliferation of these cells [33]. Studies indicate that this receptor is responsible for cell proliferation and differentiation [34,35]. In addition to the classic VDR, the cells present the 1,25D_3_-MARRS (ERp57/PDIA3), also capable of binding calcitriol and its analogs [36]. Research on mammary gland cancer cells has shown that the 1,25D_3_-MARRS protein can reduce the antiproliferative activity of calcitriol by interfering with VDR. The studies showed that both leukemia and lymphoma cells sensitive and insensitive to calcitriol and its analog proliferation inhibition have similar mRNA levels for both receptors. Most of the cell unstimulated by calcitriol and tacalcitol have a higher level of 1,25D_3_-MARRS protein compared to the VDR. The exceptions were the cells of the HL-60, K562 and KG-1 lines, which had a higher level of VDR protein. This analysis indicates that basal levels of receptors capable of binding calcitriol and tacalcitol are not responsible for the sensitivity of human leukemia and lymphoma cells to these compounds. To explain the role of the native VDR in leukemia and lymphoma cells it was necessary to determine whether single nucleotide polymorphisms exist in the VDR coding sequence that are responsible for the mRNA stability and biological activity of this protein. It was also key to clarify whether these polymorphisms are associated with varying degrees of susceptibility of leukemia and lymphoma cells to calcitriol and tacalcitol. The best described polymorphisms defined as *Fok*I, *Bsm*I, *Apa*I, and *Taq*I were selected for the research. The relationship between VDR polymorphism and cancer has not been unequivocally confirmed [17,37] as in the case of Crohn’s disease or ulcerative colitis [38,39]. The human gene encoding the VDR is located on chromosome 12, it consists of a promoter, regulatory sites (1a-1f) and exons 2–9 that encode 6 receptor protein domains (A–F) [40]. So far, several polymorphisms have been identified in the coding sequence of the VDR gene. One of them is the *Fok*I polymorphism (rs2228570), located in the second exon, which concerns the translation initiation site. The change of nucleotide from T (ATG) to C (ACG) causes the reading frame shift. As a result, the two variants of the VDR protein may arise during the translation process: the longer form of the protein (T allele or f allele) and the shorter form of the protein (C allele or F allele). It turns out that the longer form, containing 427 amino acids, is almost half as biologically active, as the shorter form is composed of 424 amino acids [15]. In 2000, Jurutka and colleagues demonstrated that the shorter 424 amino acid form of the vitamin D receptor has greater ability to bind to the TFIIB transcription factor. The shorter form of the vitamin D receptor is more transcriptionally active [41]. Study of Colin and co-workers also confirmed that the 424 amino acid form of the VDR protein has a higher transcription activity [42]. Much attention was paid to polymorphisms locating in the 3′UTR regulatory region of the VDR gene. It was found that there are several single nucleotide polymorphisms in this region, and three polymorphisms have the greatest association: *Bsm*I (rs1544410), *Apa*I (rs7975232), and *Taq*I (rs731236). It is known that the 3′UTR region is responsible for the regulation of gene expression, and especially for the stability of mRNA [43]. Using a method based on luciferase activity, differences in activity were detected between the variants of the 3′UTR region that are associated with “baT” or “BAt” haplotypes. The “baT” haplotype, which is associated with the higher translational activity of vitamin D receptor mRNA, resulting in the formation of more VDR protein in cells. However, the “baT” haplotype is defined as being more active, less stable and responsible for increased production of the vitamin D receptor protein [18,36,42,43]. Studies using meta-analysis of data on the most-studied vitamin D-VDR receptor polymorphisms: *Fok*I, *Bsm*I, *Apa*I, and *Taq*I in cancer have shown that the presence of *Bsm*I polymorphism significantly increases the risk of developing colorectal and skin cancer [44,45]. In turn, an increased risk of developing mouth and breast cancer, and reduced in the case of prostate cancer, was noted in t allele carriers of *Taq*I polymorphism. In contrast, the f allele of *Fok*I polymorphism was found in the case of ovarian and skin cancer cells, at the same time it was a factor reducing the incidence of glioblastoma [37,46,47]. The presence of *Fok*I polymorphism in the second exon encoding the VDR leads to the formation of a shorter form of a 427 but 424 amino acid protein, which is twice as active transcriptionally [15]. In turn, the remaining *Bsm*I, *Apa*I and *Taq*I polymorphisms are located in the 3′UTR region of the gene encoding the VDR and are responsible for the transcriptional activity of mRNA [44]. Analysis of human leukemia and lymphoma cells selected for testing showed that calcitriol and tacalcitol sensitive MV-4-11, Thp-1 and HL-60 cells had a single nucleotide polymorphism at the initiation site of the VDR gene translation. These cells turned out to be Ft heterozygous, which leads to the formation of two forms of vitamin D receptor protein: a longer one composed of 427 amino acids-less transcriptionally active and less responsive to calcitriol, and a shorter composed of 424 amino acids, more transcriptionally active. Jurkat cells, which were insensitive to calcitriol, also turned out to be Ft heterozygous. The presence of only the *Fok*I polymorphism determining the activity of the VDR protein is insufficient. This is confirmed by studies of the “bat” haplotype because only sensitive cells showed the presence of the “baT” haplotype in the 3′UTR region, which is responsible for the high translational activity of vitamin D receptor mRNA. These results are consistent with the analysis of vitamin D receptor protein levels because the largest increase in VDR protein after calcitriol and tacalcitol treatment was observed in MV-4-11 and Thp-1 cells. Only HL-60 cells were characterized by the presence of the “BAt” haplotype. At the same time, we observed the lower sensitivity of HL-60 cells to calcitriol and tacalcitol. Interestingly, only these cells show the presence of the “BAt” haplotype. Therefore, we know that the challenge is to better understand the impact of this specific BAt haplotype on the biological activity of vitamin D analogs, which has not been explored so far. In the remaining cell lines selected for study, the presence of recessive ff homozygotes (K562 and KG-1), dominant FF heterozygotes (Raji, Daudi, U2932), and “bat” haplotypes not related to the stability and translational activity of classic vitamin D receptor mRNA were observed. The study suggests that the presence of the f allele of *Fok*I polymorphism and the “baT” haplotype determine the sensitivity of human leukemia and lymphoma cells to calcitriol and tacalcitol. 

## 5. Conclusions

Our data showed that the level of proteins responsible for the biological activity of calcitriol and tacalcitol are insufficient to predict the response to tested compounds. Interestingly, vitamin D receptor polymorphism but not its level could be a factor that plays a role in the sensitivity of human leukemia and lymphoma cells to calcitriol and its analog. Detected polymorphisms could be used as a biomarker to select patients for therapy and improve survival outcomes. Importantly, we would like to emphasize that to confirm the obtained results, it is necessary to conduct additional tests, including in vivo models. More data are needed on the role of this polymorphism in leukemia and lymphoma patients samples. 

## Figures and Tables

**Table 1:** Characterization of tested human leukemia and lymphoma cells.

Cell Line.	Disease	FAB	Sex and Age	Doubling Time	Gene Fusion	Gene Mutation; Zygosity
**KG-1**	acute myeloid leukemia	**M0**	Male, 59Y	38 h	FGFR1OP2-FGFR1	**NRAS** (p.Gly12Asp (c.35G>A)); unspecified **TP53** (c.672+1G>A); homozygous
**K562**	chronic myeloid leukemia	**M1**	Female, 53Y	47 h	BCR-ABL1	**TP53** (p.Gln136fs*13 (c.406_407insC)); homozygous
**HL-60**	acute myeloid leukemia	**M2**	Female, 36Y	36–48 h	n/a	**TP53**, gene deletion; homozygous **CDKN2A** (p.Arg80Ter (c.238C>T) (p.Pro94Leu, c.281C>T)); homozygous **NRAS** (p.Gln61Leu (c.182A>T); heterozygous
**MV-4-11**	acute monoblastic /monocytic leukemia	**M5**	Male, 10Y	40 h	KMT2A-AFF1 MLL-AFF1 ALL-1/AF4	**FLT3**, unexplicit, internal tandem duplication; unspecified
**Thp-1**	acute monoblastic /monocytic leukemia	**M5**	Male, 1Y	60–70 h	CSNK2A1-DDX39B KMT2A-MLLT3 MLL-MLLT3 MLL-AF9	**NRAS** (p.Gly12Asp (c.35G>A)); heterozygous **TP53** (p.Arg174fs*3 (c.520_545del26)); heterozygous
**Jurkat**	precursor T-cell acute lymphoblastic leukemia	**L2**	Male, 14Y	24 h	n/a	**BAX** (p.Glu41Argfs*19 (c.121delG) and p.Glu41Glyfs*33 (c.121dupG)); heterozygous **FBXW7** (p.Arg505Cys (c.1513C>T)); heterozygous **INPP5D** (p.Gln345Ter (c.1033C>T) and (c.1097+1065_1097+1112del47)); heterozygous **MSH2** (p.Arg711Ter (c.2131C>T)); homozygous **MSH6** (p.Phe1088Serfs*2 (c.3261delC)); homozygous **NOTCH1** (p.Arg1627His (c.4880G>A)); heterozygous **TP53** (p.Arg196Ter (c.586C>T)); heterozygous
**U2932**	diffuse large B-cell lymphoma	**L2**	Female, 29Y	48–50 h	n/a	n/a
**Daudi**	Burkitt lymphoma	**L3**	Male, 16Y	30–40 h	MYC-IGH	**B2M** (p.Met1Ile (c.3G>C)); homozygous **CTNNB1** (p.Ala5_Ala80del)); homozygous **TP53** (p.Gly266Glu (c.797G>A)); heterozygous
**Raji**	Burkitt lymphoma	**L3**	Male, 11Y	24–36 h	MYC-IGH	**TP53** (p.Arg213Gln (c.638G>A) and p.Tyr234His (c.700T>C)); heterozygous

**Table 2:** The table shows alleles of human leukemia and lymphoma cells in the gene coding for the vitamin D receptor.

Cell Line	*Fok*I	*Bsm*I	*Apa*I	*Taq*I	Haplotype
leukemia					
**MV-4-11**	Ff	bb	aa	TT	baT
**Thp-1**	Ff	bb	aa	TT	baT
**HL-60**	Ff	BB	AA	Tt	BAt
**K562**	ff	Bb	Aa	Tt	BAT
**KG-1**	ff	bb	Aa	TT	bAT
lymphoma					
**Raji**	FF	Bb	AA	Tt	BAT
**Daudi**	FF	Bb	AA	TT	BAT
**Jurkat**	Ff	Bb	Aa	TT	BAT
**U2932**	FF	Bb	Aa	Tt	BAT

## Data Availability

The data presented in this study are available in this article (and Supplementary Materials).

## Supplementary Materials

The following supporting information can be downloaded at: https://www.mdpi.com/article/10.3390/cancers14020387/s1, Figure S1a: Protein levels of vitamin D-binding receptors: classical vitamin D receptor (VDR) and 1,25D3-MARRS in leukemia and lymphoma cells, Figure S1b. The mRNA level of vitamin D binding receptors: classical vitamin D receptor (VDR), and 1,25D3-MARRS in tested cell lines, Figure S1c: The protein level of vitamin D binding receptors: classical vitamin D receptor (VDR) and 1,25D3-MARRS in tested cell lines. Figure S2: Vitamin D receptor polymorphism *Fok*I(a), *Bsm*I(b), *Apq*I(c), and *Taq*I(d), Table S1: Inhibition of cell proliferation of myeloid neoplasms, Table S2: Inhibition of cell proliferation of lymphoid neoplasms.
