# Supplementary material for: Polymorphism of VDR Gene and the Sensitivity of Human Leukemia and Lymphoma Cells to Active Forms of Vitamin D
Source: Cancers (Basel). 2022 Jan 13;14(2):387. doi: 10.3390/cancers14020387 (PMC8774213; doi:10.3390/cancers14020387)
Supplement: Supplementary file 1 [file cancers-14-00387-s001.zip › cancers-1530927-supplementary.pdf]

Supplement

Cell proliferation assay

Table S1. Inhibition of cell proliferation of myeloid neoplasms. Inhibition of proliferation using 99.8% ethanol as solvent for calcitriol and tacalcitol is shown. The control of the performed experiments was cisplatin for which the following IC_{50} value was determined (mean ± standard deviation).

	MV-4-11	Thp-1	HL-60	K562	KG-1
**ethanol 99,8 % (%)**	**10,31** ± 7,62	**5,92** ± 4,19	**0,00** ± 0,00	**13,71** ± 3,03	**12,15** ± 2,89
**cisplatin (μg/ml)**	**0,36** ± 0,04	**0,26** ± 0,02	**0,29** ± 0,03	**0,68** ± 0,07	**0,28** ± 0,07

Table S2. Inhibition of cell proliferation of lymphoid neoplasms. Inhibition of proliferation using 99.8% ethanol as solvent for calcitriol and tacalcitol is shown. The control of the performed experiments was cisplatin for which the following IC_{50} values were determined (mean ± standard deviation).

	Raji	Daudi	Jurkat	U_{2932}
**ethanol 99,8 % (%)**	**1,12** ± 1,95	**6,4** ± 2,2	**5,37** ± 3,52	**0,90** ± 1,56
**cisplatin (μg/ml)**	**0,35** ± 0,04	**0,28** ± 0,06	**0,20** ± 0,09	**0,30** ± 0,09

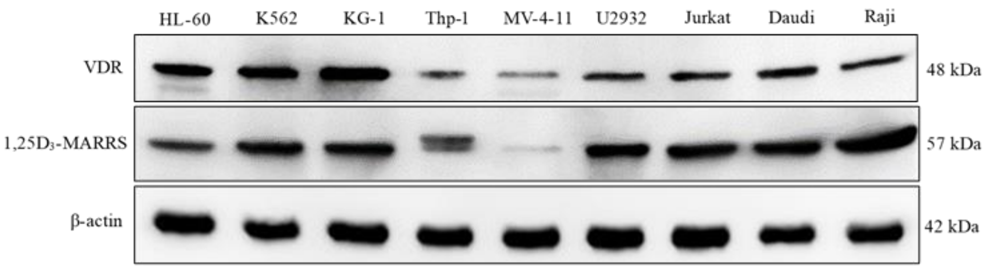

Figure S1a. Protein levels of vitamin D-binding receptors: classical vitamin D receptor (VDR) and 1,25D3-MARRS in leukemia and lymphoma cells.

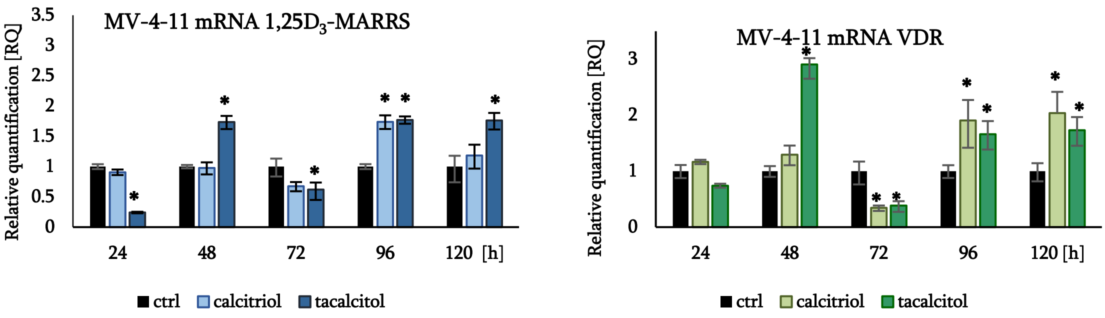

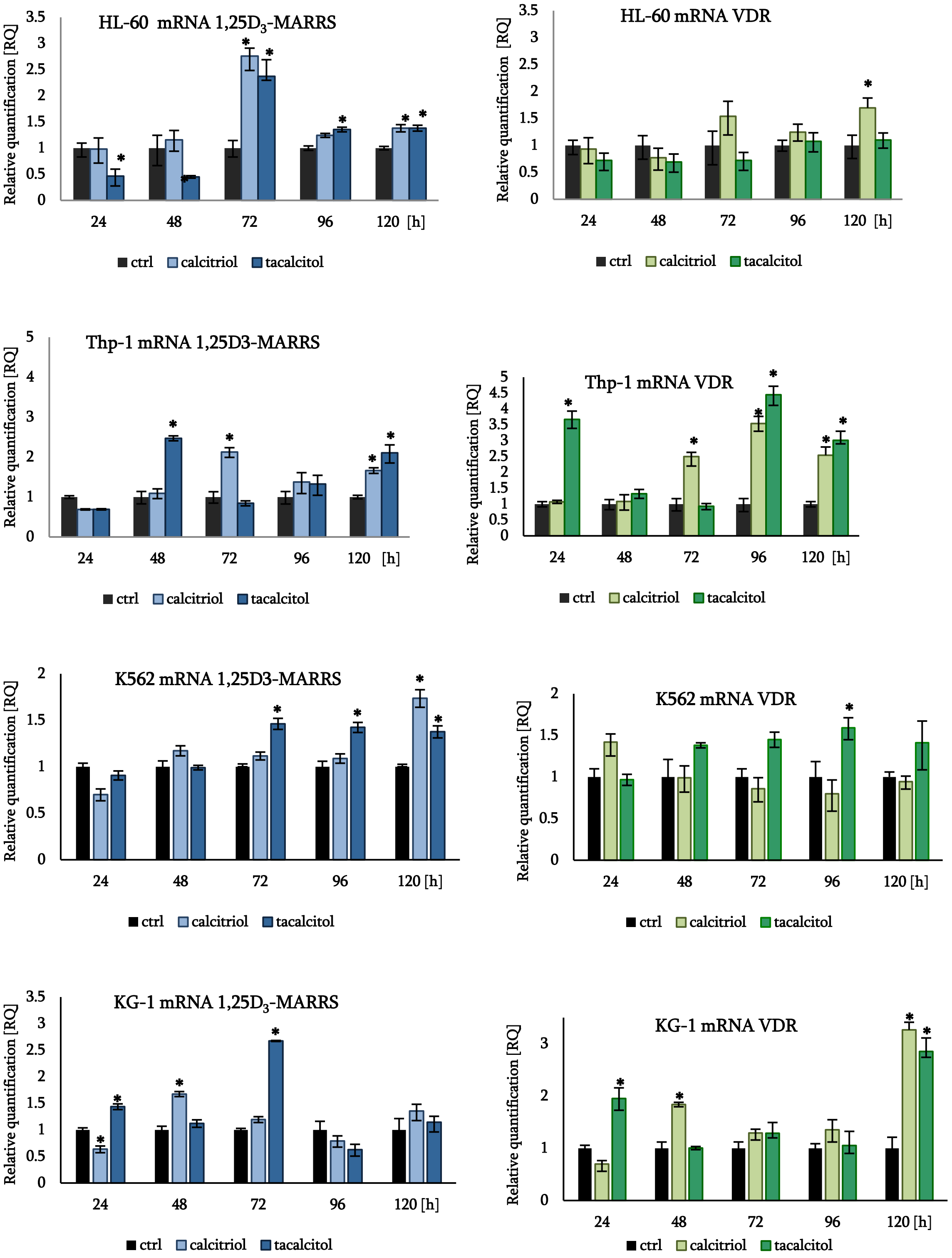

HL-60 mRNA 1,25D$_3$-MARRS
Relative quantification [RQ]
0 0.5 1 1.5 2 2.5 3 3.5
24 48 72 96 120 [h]
ctrl calcitriol tacalcitol
HL-60 mRNA VDR
Relative quantification [RQ]
0 0.5 1 1.5 2 2.5 3 3.5
24 48 72 96 120 [h]
ctrl calcitriol tacalcitol
Thp-1 mRNA 1,25D3-MARRS
Relative quantification [RQ]
0 1 2 3 4 5
24 48 72 96 120 [h]
ctrl calcitriol tacalcitol
Thp-1 mRNA VDR
Relative quantification [RQ]
0 0.5 1 1.5 2 2.5 3 3.5 4 4.5 5
24 48 72 96 120 [h]
ctrl calcitriol tacalcitol
K562 mRNA 1,25D3-MARRS
Relative quantification [RQ]
0 0.5 1 1.5 2
24 48 72 96 120 [h]
ctrl calcitriol tacalcitol
K562 mRNA VDR
Relative quantification [RQ]
0 0.5 1 1.5 2
24 48 72 96 120 [h]
ctrl calcitriol tacalcitol
KG-1 mRNA 1,25D$_3$-MARRS
Relative quantification [RQ]
0 0.5 1 1.5 2 2.5 3 3.5
24 48 72 96 120 [h]
ctrl calcitriol tacalcitol
KG-1 mRNA VDR
Relative quantification [RQ]
0 1 2 3
24 48 72 96 120 [h]
ctrl calcitriol tacalcitol

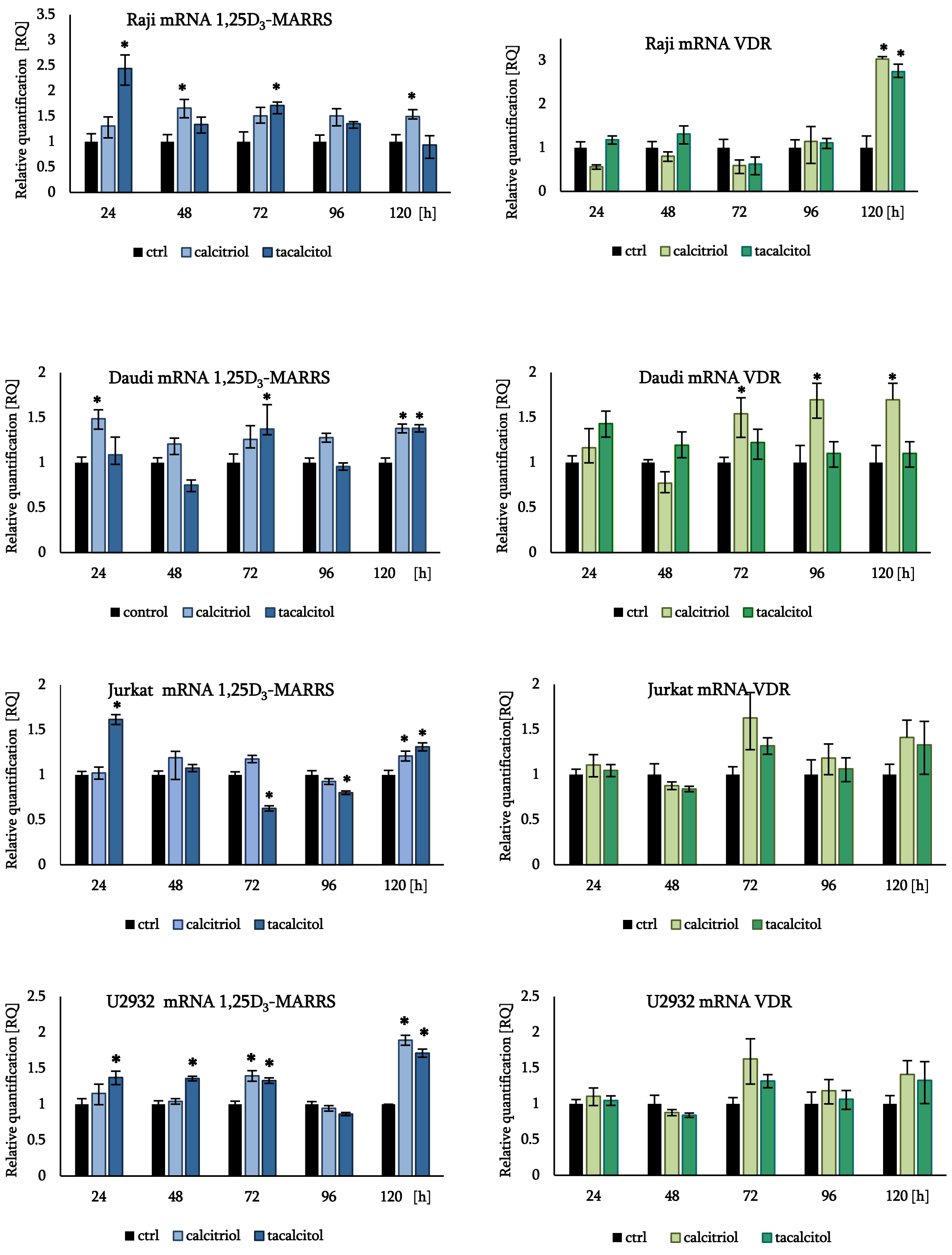

Figure S1b. The mRNA level of vitamin D binding receptors: classical vitamin D receptor (VDR), and 1,25D3-MARRS in tested cell lines. * - statistical significance ($p < 0.05$) in relation to the control from a given time point.

MV-4-11

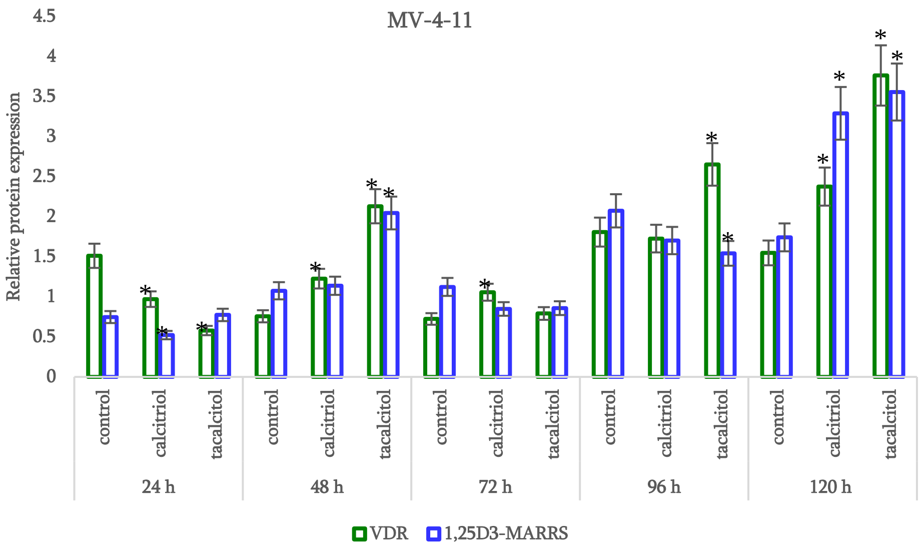
MV-4-11
Relative protein expression
0
0.5
1
1.5
2
2.5
3
3.5
4
4.5
control
calcitriol
tacalcitol
24 h
48 h
72 h
96 h
120 h
VDR
1,25D3-MARRS

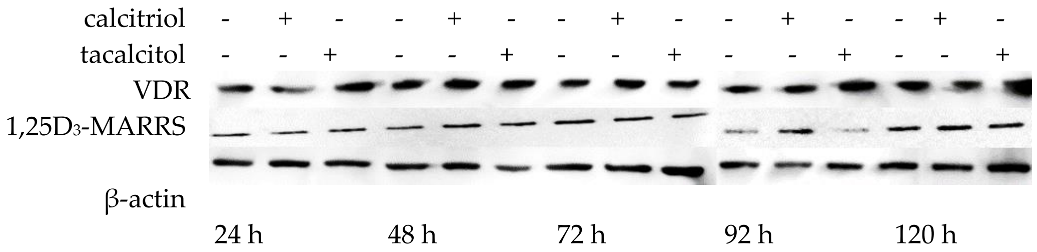
calcitriol - + - - + - - + - - + - - + -
tacalcitol - - + - - + - - + - - + - - +
VDR
1,25D3-MARRS
β-actin
24 h
48 h
72 h
92 h
120 h

THP-1

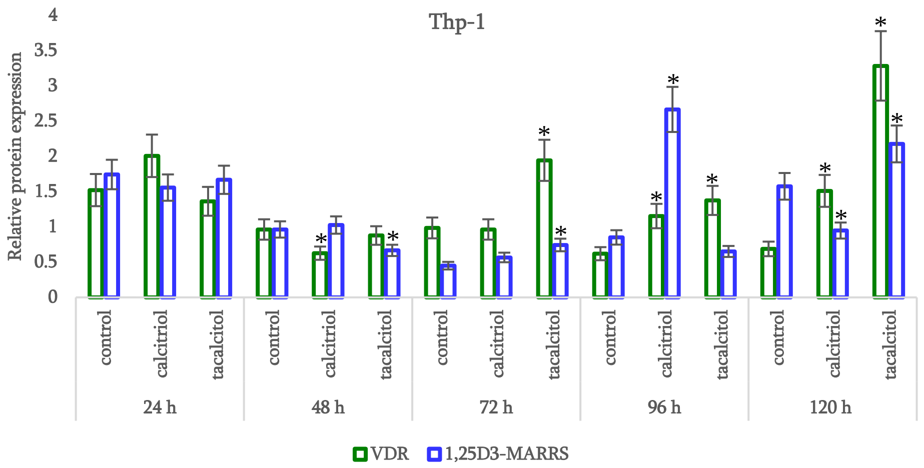
Thp-1
Relative protein expression
0
0.5
1
1.5
2
2.5
3
3.5
4
control
calcitriol
tacalcitol
24 h
48 h
72 h
96 h
120 h
VDR
1,25D3-MARRS

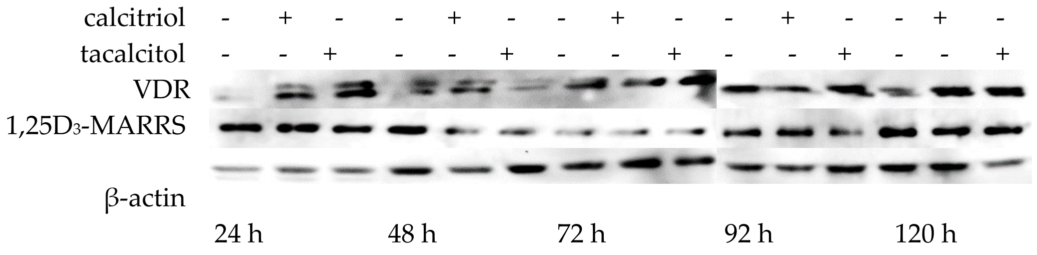
calcitriol - + - - + - - + - - + - - + -
tacalcitol - - + - - + - - + - - + - - +
VDR
1,25D3-MARRS
β-actin
24 h
48 h
72 h
92 h
120 h

HL-60

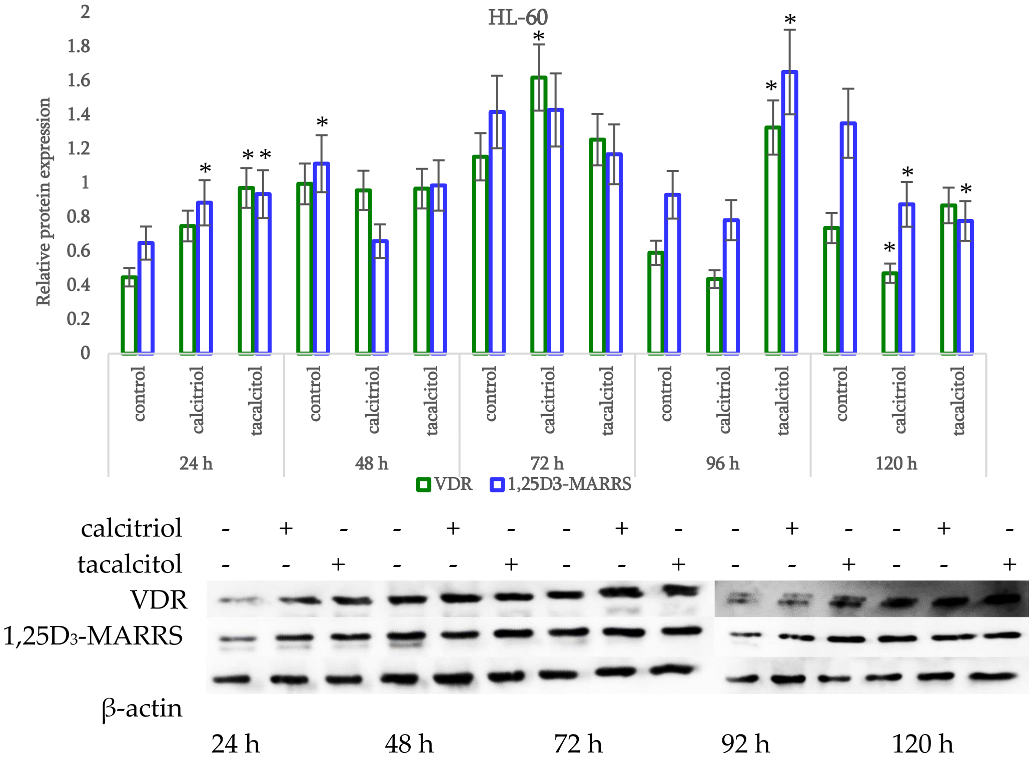
HL-60
Relative protein expression
control
calcitriol
tacalcitol
24 h
48 h
72 h
96 h
120 h
VDR
1,25D3-MARRS
calcitriol
tacalcitol
VDR
1,25D3-MARRS
β-actin
24 h
48 h
72 h
92 h
120 h

K562

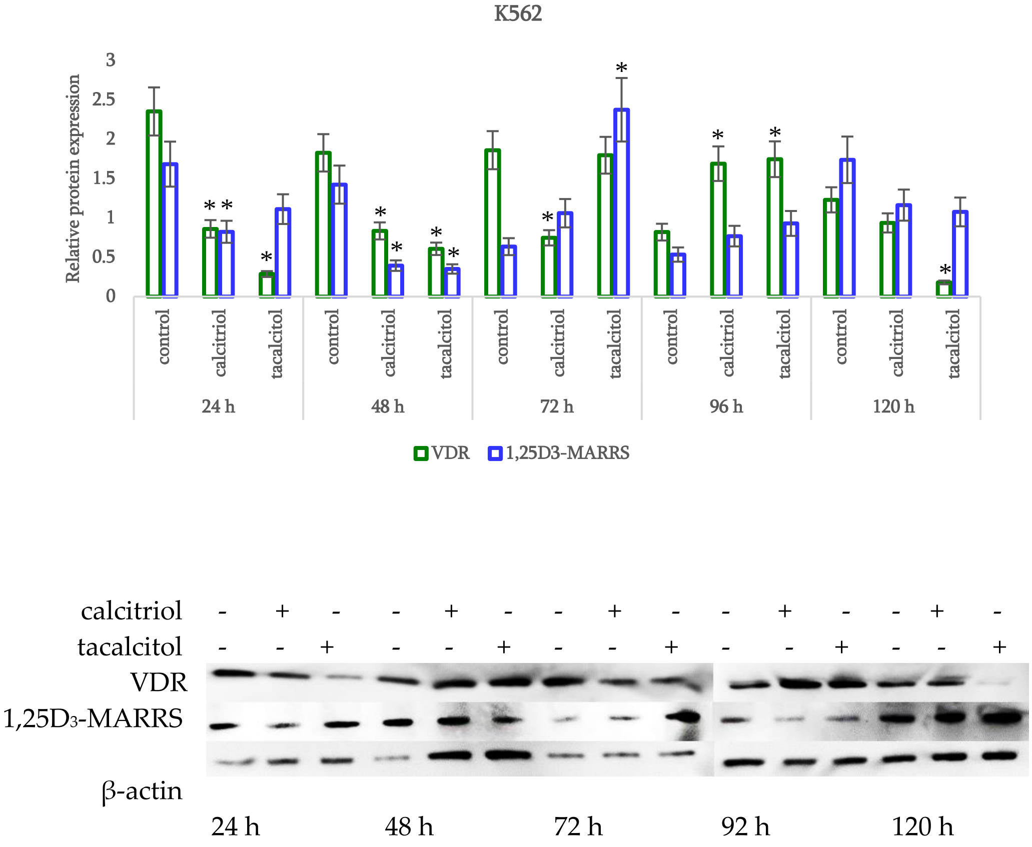
K562
Relative protein expression
control
calcitriol
tacalcitol
24 h
48 h
72 h
96 h
120 h
VDR
1,25D3-MARRS
calcitriol
tacalcitol
VDR
1,25D3-MARRS
β-actin
24 h
48 h
72 h
92 h
120 h

KG-1

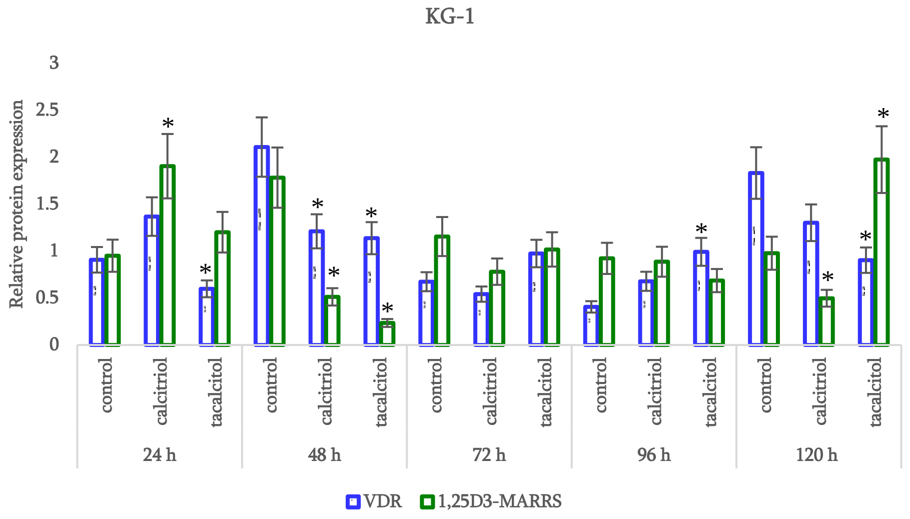
KG-1
Relative protein expression
0
0.5
1
1.5
2
2.5
3
control
calcitriol
tacalcitol
24 h
48 h
72 h
96 h
120 h
VDR
1,25D3-MARRS

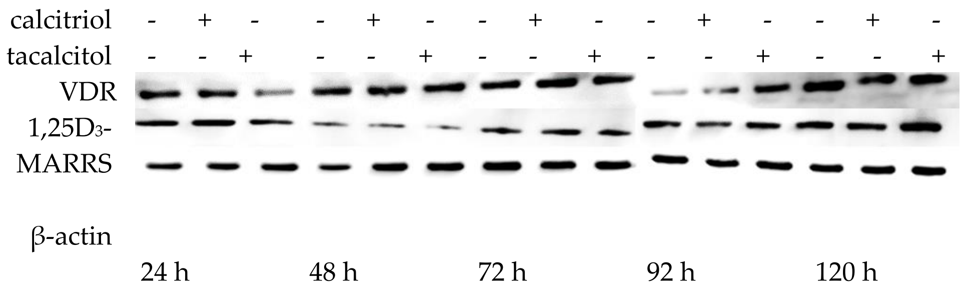
calcitriol - + - - + - - + - - + - - + -
tacalcitol - - + - - + - - + - - + - - +
VDR
1,25D3-
MARRS
β-actin
24 h
48 h
72 h
92 h
120 h

Raji

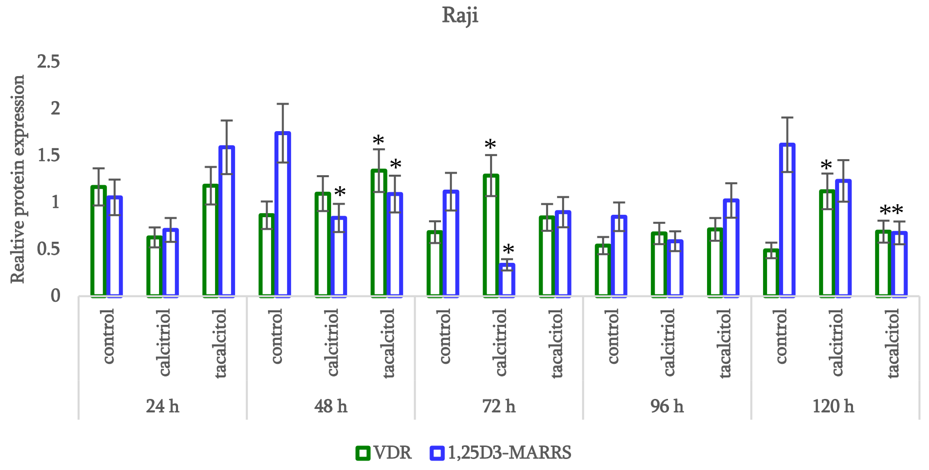
Raji
Realtive protein expression
0
0.5
1
1.5
2
2.5
control
calcitriol
tacalcitol
24 h
48 h
72 h
96 h
120 h
VDR
1,25D3-MARRS

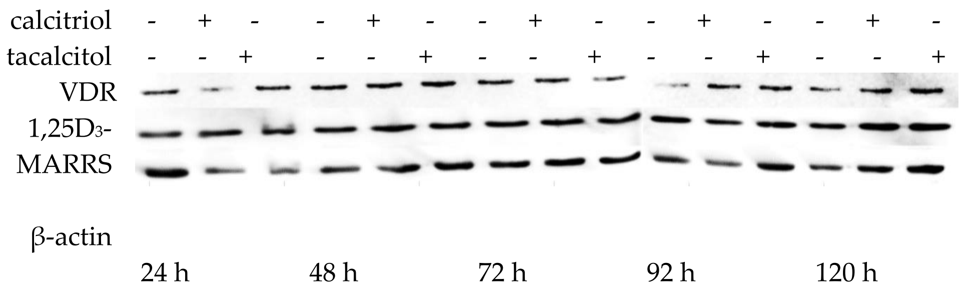
calcitriol - + - - + - - + - - + - - + -
tacalcitol - - + - - + - - + - - + - - +
VDR
1,25D3-
MARRS
β-actin
24 h
48 h
72 h
92 h
120 h

Daudi

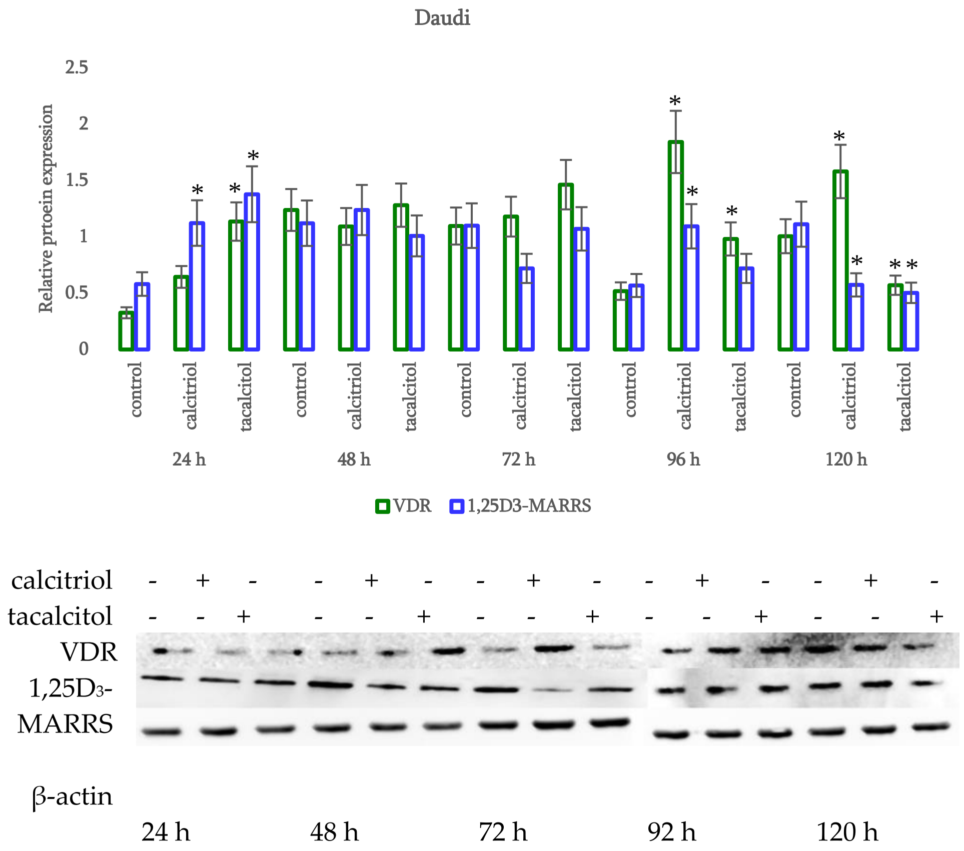

Jurkat

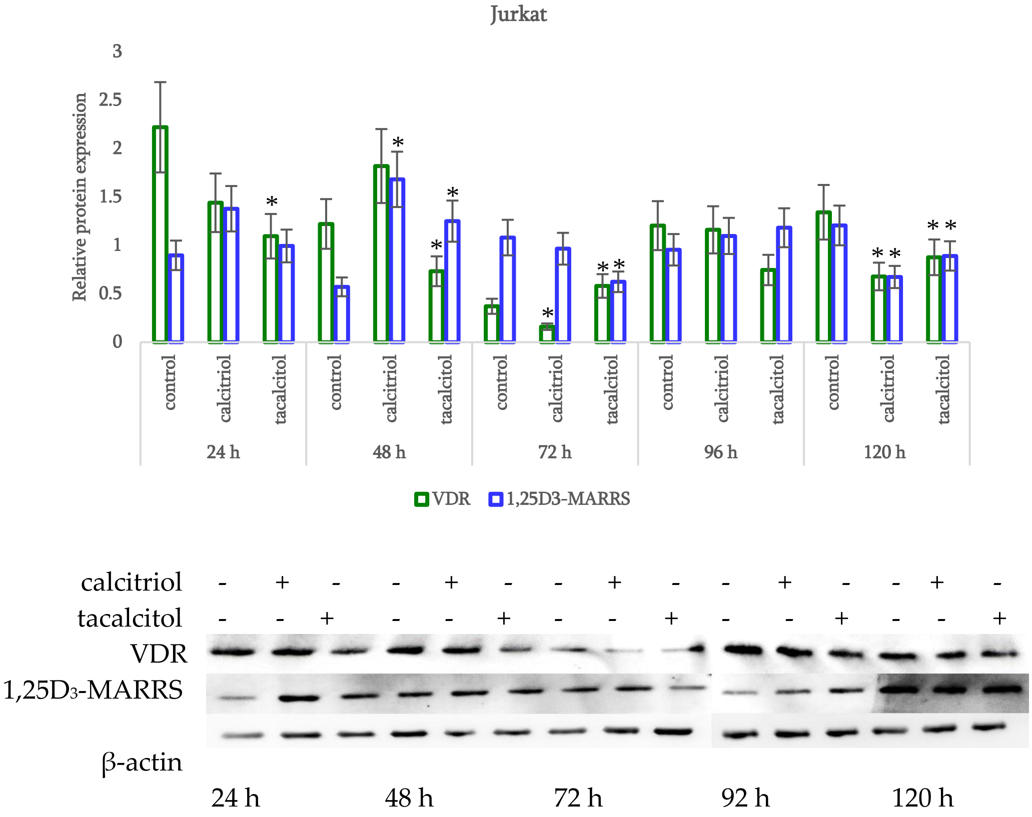

U2932

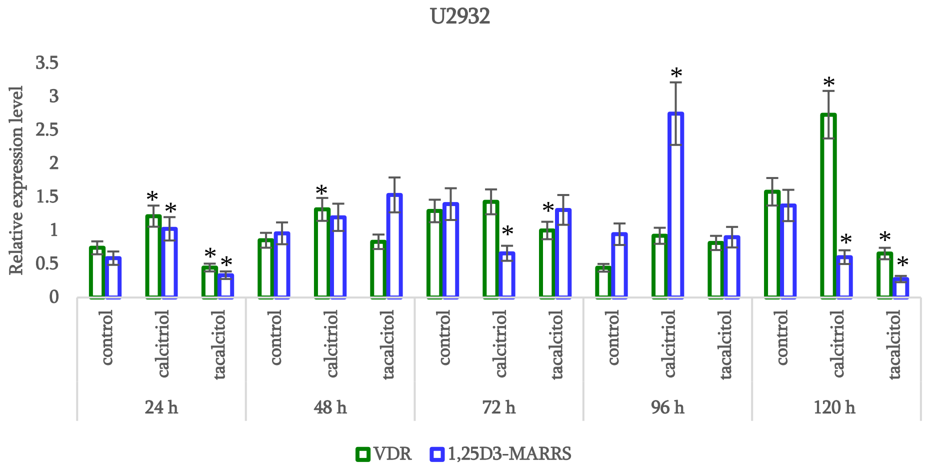
U2932
Relative expression level
3.5
3
2.5
2
1.5
1
0.5
0
control
calcitriol
tacalcitol
24 h
48 h
72 h
96 h
120 h
VDR
1,25D3-MARRS

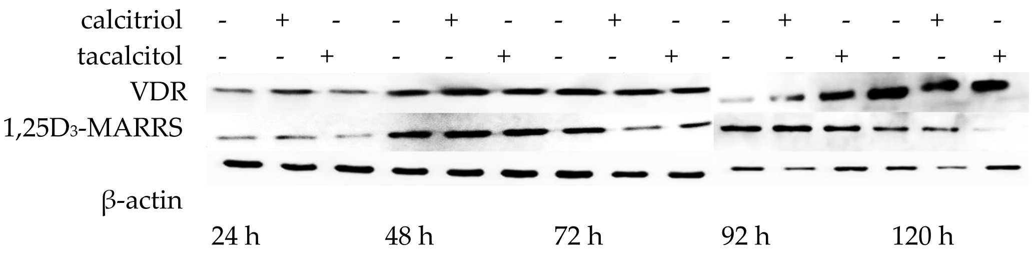
calcitriol
tacalcitol
VDR
1,25D3-MARRS
β-actin
24 h
48 h
72 h
92 h
120 h

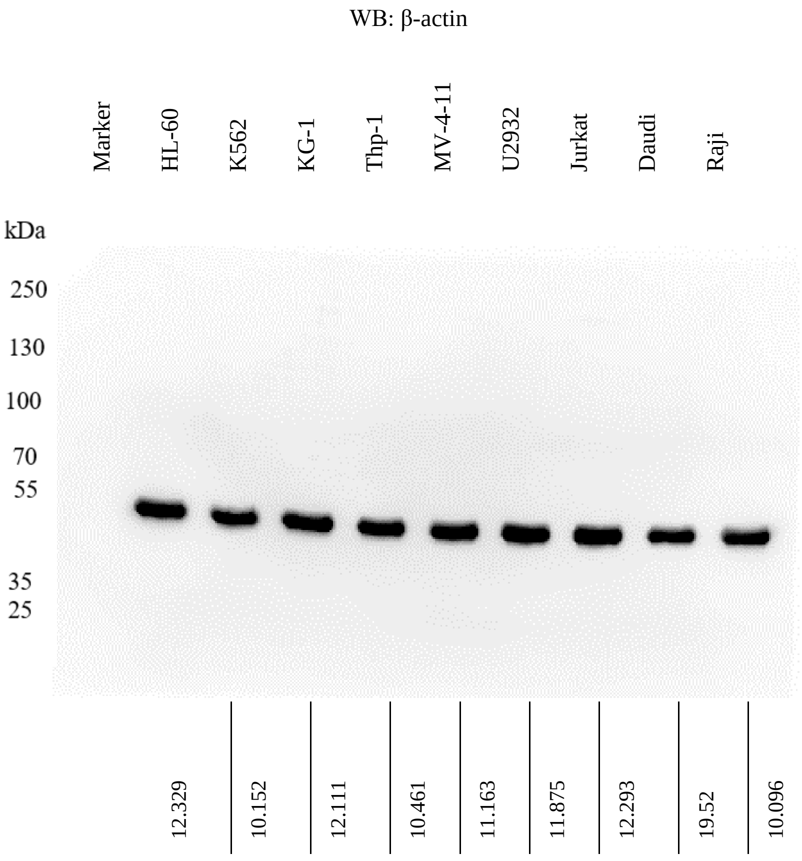
WB: β-actin
Marker
HL-60
K562
KG-1
Thp-1
MV-4-11
U2932
Jurkat
Daudi
Raji
kDa
250
130
100
70
55
35
25
12.329
10.152
12.111
10.461
11.163
11.875
12.293
19.52
10.096

WB: VDR

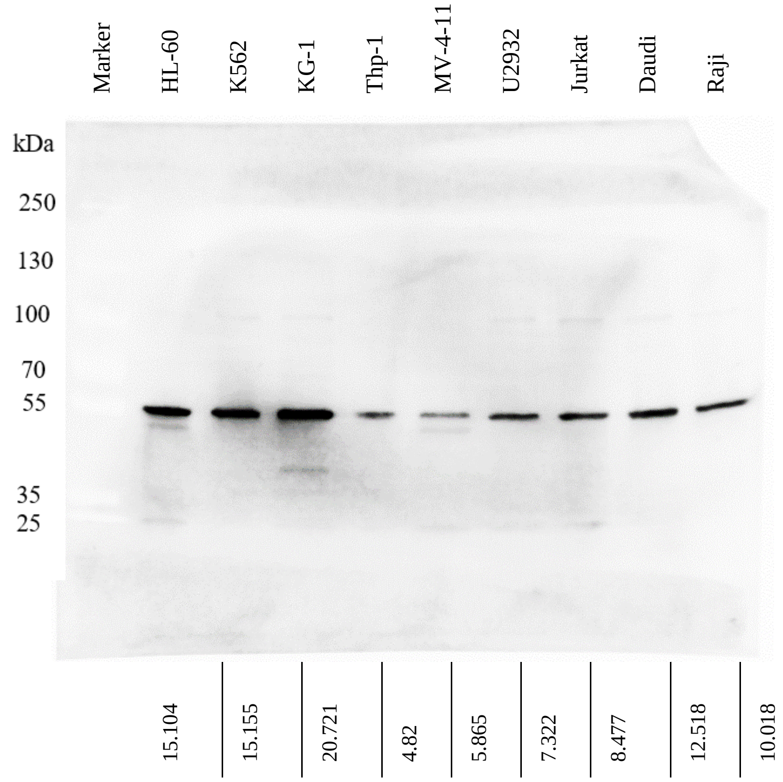

WB: 1,25D3-MARRS

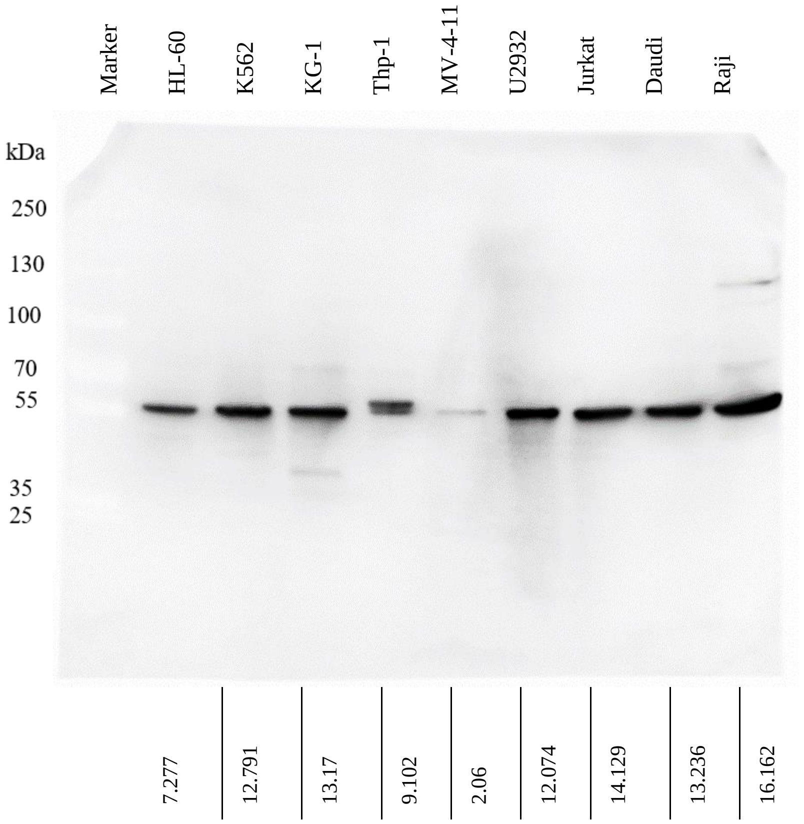

Figure S1c. The protein level of vitamin D binding receptors: classical vitamin D receptor (VDR) and 1,25D3-MARRS in tested cell lines. The relative level of test proteins was estimated by densitometric

measurement of the analyzed protein signal obtained by Western blotting, which was normalized to the β-actin control signal (representative of the results from the Western blot). * - statistical significance ($p < 0.05$) in relation to the control from a given time point. Raw western blot with marker for each presented protein was shown at the end.

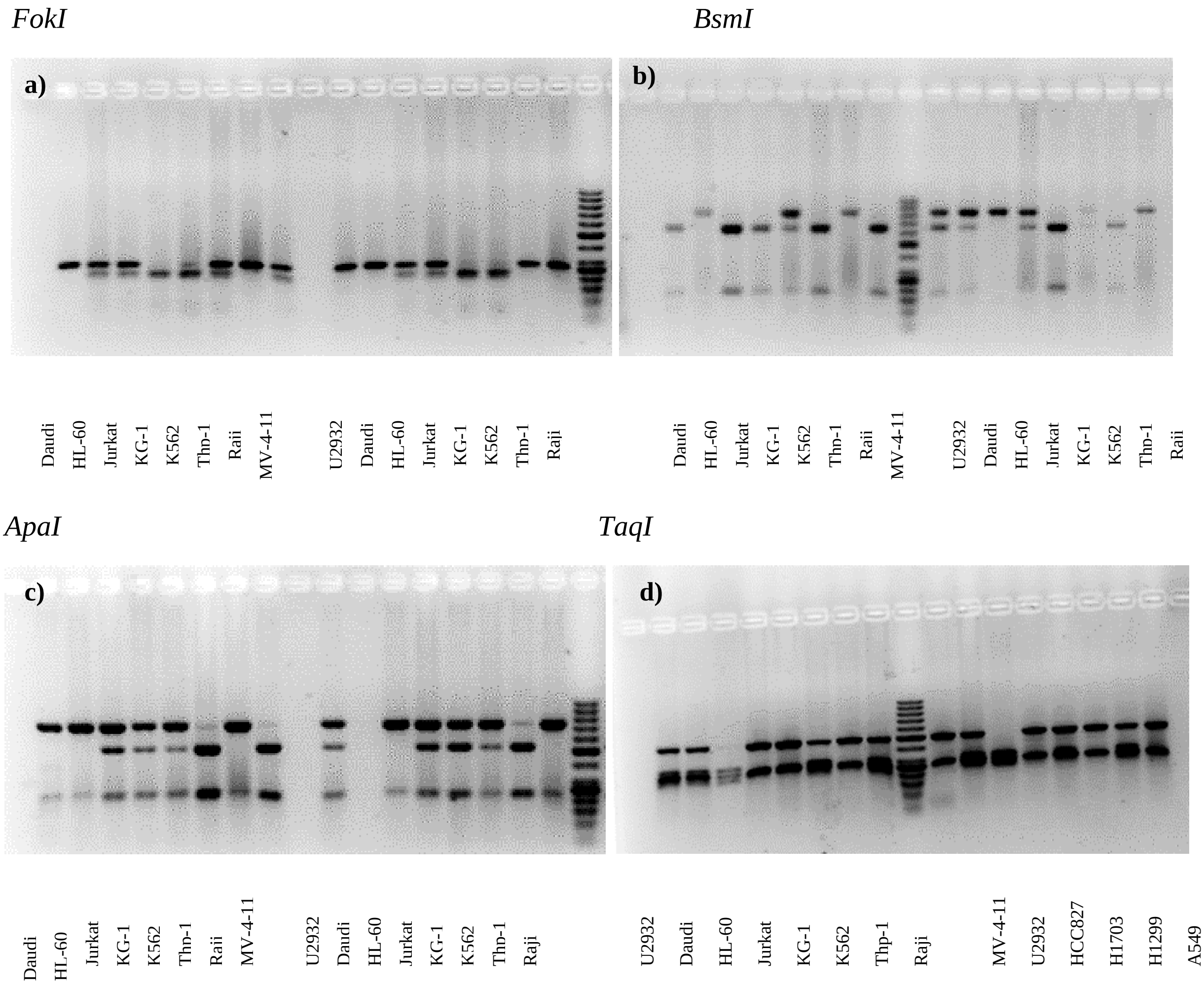

Figure S2. Vitamin D receptor polymorphism *FokI(a), BsmI(b), ApqI(c)*, and *TaqI(d)*

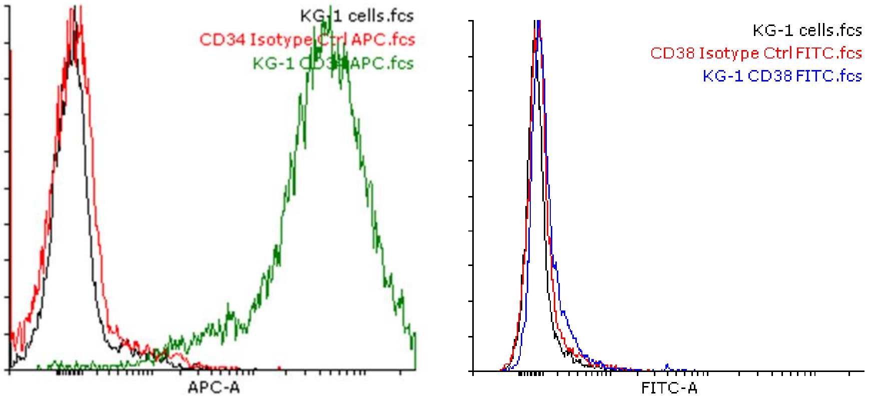

Figure S3. Surface markers CD34 and CD38 in KG-1 cell line. Control unstained KG-1 cells (black), CD34 isotype control APC antibody (left red), CD38 isotype control FITC antibody (right red), KG-1 cells with anti-CD34 APC antibody (green), KG-1 cells with anti-CD38 FITC antibody (blue).
